# Oleogel Dressings for Skin Therapy: Physicochemical and Bioactive Properties of Cosmetic Oil-Based Systems Enriched with Essential Oils

**DOI:** 10.3390/gels12030248

**Published:** 2026-03-15

**Authors:** Andres Zapata Betancur, Freddy Forero Longas, Adriana Pulido Diaz

**Affiliations:** 1 Pharmacy Department, University of Antioquia UdeA, Calle 67, 53-108, Medellin 050010, Colombia; andres.zapatab@udea.edu.co; 2 Food Engineering Department, University of Antioquia UdeA, Calle 67, 53-108, Medellin 050010, Colombia; 3 Agrosavia, La Selva Research Center, Rionegro 055038, Colombia; apulido@agrosavia.co

**Keywords:** skin recovery, natural waxes, bactericidal activity, keratinocyte proliferation

## Abstract

Developing potential skincare formulations capable of simultaneously managing infection and promoting tissue repair remains a critical challenge in dermatological care. This study engineered bioactive oleogels using sunflower wax (SFW), rice bran wax (RBW), and 12-hydroxystearic acid (HSA) to deliver a synergistic essential oil blend (ginger, cinnamon, tea tree, geranium). A D-optimal mixture design optimized formulations to match the textural profile of a commercial benchmark. Crucially, the fatty acid architecture of the carrier oil emerged as a primary determinant of network integrity; the high oleic acid content in camellia oil facilitated robust RBW crystallization by minimizing steric hindrance, whereas the polyunsaturated, kinked structure of linoleic acid in almond oil disrupted SFW networks, resulting in lower stiffness. Thermal characterization (DSC) established a distinct stability hierarchy with RBW exhibiting the highest melting point (Tp = 60.1 °C) and enthalpy (ΔHm = 7.79 ± 0.74 J/g). Thermogravimetric analysis (TGA) confirmed high thermal resistance for wax-based systems (Tdeg ≈ 357 °C), whereas HSA displayed a biphasic degradation starting at ~206 °C. FTIR spectroscopy verified the stable physical entrapment of bioactives, with the lipid vehicle dominating the spectral fingerprint. Rheological profiling revealed that RBW oleogels, structured in high-oleic camellia oil, formed rigid networks (G′ ≈ 5.7 × 104 Pa) with high yield stress (20.91 Pa), offering superior retention. In contrast, HSA oleogels displayed “smart” thixotropic recovery with lower stiffness (G′ ≈ 2.1 × 104 Pa) and a distinct melting peak at 22.5 °C, compared to 60.1 °C for RBW. All formulations achieved a >2 Log_10_ reduction (99%) in *Staphylococcus aureus* and *Pseudomonas aeruginosa* viability after 12 h. Furthermore, in vitro keratinocyte assays identified a hormetic therapeutic window at 1–5 μg/mL (essential oil blend equivalent); specifically, SFW oleogels at 5 μg/mL stimulated proliferation to 158.07% relative to controls. These findings confirm that optimizing the lipid vehicle–bioactive interface creates dual-action scaffolds capable of simultaneously managing infection and stimulating in vitro keratinocyte proliferation.

## 1. Introduction

Cutaneous wound healing, an orchestrated biological process, is fundamental for maintaining skin integrity, the body’s primary defense. An intricate sequence comprises four overlapping phases: hemostasis, inflammation, proliferation, and remodeling. Following injury, hemostasis begins, featuring vasoconstriction and fibrin clot formation, which creates a provisional matrix for infiltrating cells [1]. A subsequent inflammatory phase involves recruiting neutrophils and macrophages, which are crucial for clearing debris, phagocytosing bacteria, and releasing signaling molecules, including pro-inflammatory cytokines like tumor necrosis factor-alpha (TNF-α) and interleukin-1β (IL-1β), and growth factors like platelet-derived growth factor [2]. The inflammatory milieu stimulates fibroblasts to proliferate and migrate into the wound bed, depositing a collagen-rich extracellular matrix (ECM), while angiogenesis restores vascular supply and keratinocytes re-epithelialize the wound surface [3]. A final remodeling phase can last months or years, during which ECM is reorganized, and type III collagen is replaced by stronger type I collagen, ultimately increasing new tissue tensile strength [4]. While robust, healing cascade progression depends upon a delicate balance of these cellular and molecular events. Disruption at any stage can cause impaired healing and chronic wound development, a major morbidity source and a significant global healthcare challenge [5]. Pathological conditions such as a prolonged inflammatory phase, excessive matrix metalloproteinase levels that degrade ECM, and reduced growth factor bioavailability can stall a wound in a non-healing state [6]. Primary contributors to this pathological state are microbial colonization and biofilm formation—structured communities of bacteria like *Staphylococcus aureus* and *Pseudomonas aeruginosa* that highly resist conventional antibiotics and host defenses [7]. Furthermore, systemic comorbidities, notably diabetes mellitus and peripheral vascular disease, create a hostile local environment of hypoxia, ischemia, and neuropathy, severely compromising the body’s intrinsic repair capacities [8]. Addressing these multifaceted barriers—infection, persistent inflammation, and poor tissue perfusion—is a central goal in modern wound care.

Wound dressing evolution reflects a growing understanding of complex healing biology. Historically, traditional dressings made from materials like cotton gauze and lint were used primarily for their absorptive capacity and for providing physical coverings. These passive materials are now considered suboptimal; they often adhere to the wound bed, causing trauma upon removal, and their fibers can act as foreign bodies [9]. Moreover, they fail to provide the moist microenvironment now considered the care standard. A paradigm shift towards moist wound healing, established by George Winter’s seminal 1960s work, demonstrated that maintaining wound interface hydration significantly accelerates re-epithelialization and reduces scar formation, a foundational principle that continues guiding modern wound care product development [10]. A new generation of advanced dressings includes diverse materials such as polymeric films, foams, hydrocolloids, and hydrogels, each with specific indications and limitations. Polyurethane films are transparent and conformable but have limited absorptive capacity, while foams are highly absorbent but opaque [11]. Hydrogel dressings are valued for donating moisture to dry, necrotic wounds, providing a cooling effect; however, they often exhibit poor mechanical strength and low adhesiveness and may require a secondary dressing for security [12]. Hydrocolloids are highly occlusive and effective at promoting autolytic debridement, but their non-transparent nature complicates wound inspection, and their complete occlusion can promote anaerobic bacteria growth [13]. Critically, a common limitation across many platforms, especially aqueous-based hydrogels, is their inefficiency in incorporating and delivering lipophilic (oil-based) therapeutic agents, which are often poorly soluble in a hydrated polymer network. This significant limitation hinders their ability to deliver a vast array of natural and synthetic active compounds.

As a versatile alternative, oleogels have garnered significant interest as advanced platforms for topical and transdermal applications. Oleogels, or organogels, are semi-solid systems with a continuous lipophilic phase, such as vegetable or cosmetic oil, immobilized within a three-dimensional network formed by a low organogelator concentration [14]. These gelators can be diverse, from natural waxes and fatty acids to synthetic polymers like ethylcellulose, allowing physicochemical property fine-tuning, including texture, hardness, and viscosity [15]. Gel network formation relies on non-covalent interactions, including hydrogen bonding and van der Waals forces, resulting in a thermo-reversible system with excellent physical stability. Lipid components’ inherent biocompatibility, often including oils rich in essential fatty acids, makes them highly suitable for direct application to compromised skin [16]. Oleogels’ unique physicochemical characteristics make them well-suited for wound dressing applications. Their primary advantage is their occlusive nature; by forming a hydrophobic barrier on the skin surface, they reduce transepidermal water loss, preventing wound desiccation, minimizing scab formation, and facilitating keratinocyte migration [17]. An occlusive barrier also protects the wound from external contaminants and secondary infections. Furthermore, the continuous lipid phase solubilizes and sustainably releases lipophilic active pharmaceutical ingredients, a critical advantage over aqueous systems [18]. The oil phase’s inherent emollient properties also soothe and hydrate the surrounding skin, reducing itching and discomfort. Their rheological and textural property tunability allows designing formulations that are easy to apply, conformable to the wound bed, and removable without causing trauma [14,19].

Oleogel platform therapeutic efficacy can be substantially amplified by incorporating essential oils, which are complex, volatile mixtures of natural phytochemicals renowned for broad pharmacological profiles. Sourced from various aromatic plant parts, these oils contain hundreds of compounds, primarily terpenes (e.g., monoterpenes, sesquiterpenes) and phenylpropanoids, which synergistically exert a therapeutic effect [20]. For centuries, essential oils were integral to traditional medicine for treating skin ailments; modern science now validates these uses by elucidating their action mechanisms. For wound healing, their most relevant properties include potent antimicrobial, anti-inflammatory, antioxidant, and tissue-regenerative activities [21]. Their pleiotropic functionality allows them to address multiple pathological factors in a wound simultaneously, a significant advantage over single-target synthetic drugs. Specific essential oils demonstrate remarkable efficacy in preclinical wound repair models; by targeting infection, inflammation, and cellular proliferation, these natural agents offer a multi-pronged approach to supporting the endogenous healing cascade. Tea tree (*Melaleuca alternifolia*) oil, rich in terpinen-4-ol, offers broad-spectrum antimicrobial action by disrupting microbial cell membranes and inhibiting respiration [22]. Its efficacy against bacteria like *Cutibacterium acnes*, the primary causative agent of acne, and opportunistic pathogens such as *Staphylococcus aureus* positions it as a key agent [23]. This mechanism, which increases membrane permeability and causes leakage of essential metabolites, ultimately leads to cell death [24]. Similarly, geranium (*Pelargonium asperum*) essential oil exhibits significant therapeutic potential [25]. Its documented anti-inflammatory, antioxidant, and antibacterial effects are valuable for skin health [26,27]. Geranium oil helps control inflammatory diseases by curbing pro-inflammatory cytokines [28] and is effective against skin-infecting fungi [29]. Its strong safety profile establishes it as a practical choice for long-term wound care [30].

Cinnamon (*Cinnamomum cassia*) essential oil serves as a multifunctional agent for wound healing by reducing inflammation, enhancing collagen formation, and promoting cell proliferation [31]. Additionally, its main component, trans-cinnamaldehyde, provides neuroprotective effects [32], and the oil displays potent antifungal and antibacterial capabilities against food spoilage organisms and clinical pathogens [33,34]. Ginger (*Zingiber officinale*) essential oil has a long history of use for its antioxidant properties [35,36]. Research confirms its antimicrobial effects against oral and foodborne bacteria [37]. While its bioactive component, 6-gingerol, has been investigated for wound healing benefits, its practical application is challenged by unfavorable pharmacokinetic traits, including poor aqueous solubility and low bioavailability [38]. Strategically combining an oleogel vehicle with bioactive essential oils represents a promising approach for developing next generation skin dressings, and the resulting synergy leverages the beneficial properties of both components: oleogel provides a protective, hydrating, and occlusive microenvironment while acting as a stable delivery system, and essential oils provide potent, multi-target therapeutic activity to combat infection and modulate inflammation [39,40]. Preliminary studies offer proof of concept for this approach, demonstrating successful formulation of oleogels containing essential oils like tea tree or *Lantana camara* with favorable stability and antimicrobial properties [41,42]. These early findings underscore such systems’ potential to create a supportive microenvironment that favors cellular proliferation and targets microbial colonization.

Comprehensive research that systematically correlates detailed physicochemical attributes—such as rheological behavior, texture profile analysis, and stability—with a broad, therapeutic relevant spectrum of bioactive functionalities is distinctly lacking. Therefore, the present study fills a knowledge gap by systematically correlating the physicochemical properties (rheology, thermal behavior) of cosmetic oil-based oleogels with their in vitro bioactive performance. Among the diverse range of natural structuring agents, sunflower wax (SFW), rice bran wax (RBW), and 12-hydroxystearic acid (HSA) were selected for this study due to their high gelling efficiency at low concentrations and their distinct crystallization mechanisms—ranging from platelet-like (SFW) and needle-like (RBW) crystals to self-assembled fibrillar networks (HSA)—which allows for a comprehensive evaluation of how the microstructural architecture influences the bioactive profile. Associating physicochemical structure with bioactive performance provides a scientific basis for rationally designing advanced, effective, and natural-based oleogel dressings for skin healing.

## 2. Results and Discussion

### 2.1. Vegetable Oils

Characterizing the lipid feedstock fatty acid profile is foundational to designing novel structured systems like oleogels; this composition can be used to predict the final product’s physicochemical and functional properties—including structural network, rheology, oxidative stability, and therapeutic potential—because it is governed by the proportions of saturated, monounsaturated, and polyunsaturated fatty acids. Table 1 shows the distinct fatty acid profiles of almond and camellia oils to rationalize their specific applications. Almond oil contains high unsaturated fatty acids (90.591 g/100 g), balanced between monounsaturated (MUFA; 66.820 g/100 g) and polyunsaturated (PUFA; 23.771 g/100 g) fatty acids. Oleic acid predominates, followed by linoleic acid, yielding an oleic/linoleic (O/L) ratio of 2.790. In contrast, camellia oil, also rich in unsaturated fatty acids, has a profile skewed toward monounsaturated. Its high MUFA content consists mainly of oleic acid; consequently, its PUFA content is nearly threefold lower than almond oil’s, yielding a high O/L ratio of 9.261. The differing degree of unsaturation determines the oils’ susceptibility to oxidation. The Cox index, a value calculated from mono- and polyunsaturated fatty acid percentages, quantitatively predicts this susceptibility; a lower index signifies greater stability [43]. The data confirms a stark contrast: camellia oil’s low Cox index of 1.71 denotes high stability, whereas almond oil’s higher index of 3.12 indicates that it is significantly more prone to oxidation.

The dermatological effects of these oils depend on the biological roles of their constituent fatty acids; oleic acid acts as a penetration enhancer by fluidizing the lipid matrix of the stratum corneum, whereas linoleic acid serves as a direct, structural precursor to ceramide 1, a lipid critical for repairing and maintaining the epidermal barrier. Oleic acid, an omega-9 fatty acid, is the primary component in both oils (almond: 66.332 g/100 g; camellia: 78.750 g/100 g) and acts as an emollient and penetration enhancer; by fluidizing the stratum corneum’s lipid matrix, it increases the permeation of other bioactive compounds [44]. In wound healing, it stimulates pro-inflammatory action during initial tissue repair, which is essential for clearing debris and initiating healing [45]. Linoleic acid, an essential fatty acid, is abundant in almond oil but is a smaller fraction in camellia oil. This concentration difference is significant, as linoleic acid is a direct precursor to ceramide 1, a critical component of the epidermal barrier. Its deficiency compromises the barrier and can cause scaly dermatitis. The potent anti-inflammatory properties of linoleic acid help resolve initial inflammation during wound healing, making oils rich in it beneficial for chronic inflammatory skin conditions [46]. Saturated fatty acids (SFAs), though minor components, are integral to skin structure. Total SFA content was higher in camellia oil than in almond oil, and this fraction, mainly palmitic and stearic acids, contributes to the skin barrier’s structural integrity, as both are key precursors of stratum corneum lipids like ceramides. The distinct balance of saturated and unsaturated fatty acids in each oil dictates their specific effects on maintaining a resilient epidermal barrier [47].

The measured fatty acid contents for camellia oil and almond oil fall within reported concentration ranges [48,49]. Camellia oil’s high intrinsic oxidative stability, conferred by its high oleic acid and low PUFA content and validated by a low Cox index of 1.71, together with its capacity to upregulate filaggrin expression, substantiates its therapeutic application for atopic dermatitis [50]. Conversely, almond oil’s balanced oleic–linoleic profile underpins its emollient properties. While its higher PUFA content reduces its oxidative stability (Cox index 3.12), its lipid matrix effectively carries its key bioactive, α-tocopherol, contributing to its efficacy in anti-aging and photoprotection [51]. These findings are in strong concordance with the existing research, which delineates the unique dermatological niches for each oil based on their composition.

Camellia oil’s slightly higher saturated fatty acid content may contribute to a firmer, more robust oleogel network. Its low PUFA content confers high oxidative stability (Cox index 1.71), a critical attribute for topical formulations because cutaneous lipid peroxidation can generate reactive oxygen species, causing cellular damage and inflammation [52]. Oleogels containing camellia oil would exhibit longer shelf-life and lower risk of inducing oxidative stress. A camellia-based oleogel can act as a dermatological vehicle because its mechanism surpasses simple occlusion; filaggrin upregulation helps to address atopic dermatitis pathophysiology [50]. The anti-inflammatory activity of its unsaponifiable components (triterpenes, polyphenols) further supports this mechanism by downregulating pro-inflammatory mediators like iNOS (Inducible Nitric Oxide Synthase) and COX-2 (Cyclooxygenase-2) in cellular models [53]. This dual action—enhancing barrier function while suppressing inflammation—makes camellia oil ideal for advanced therapeutic skincare. Almond oil, a bioactive thinner, offers different properties because its balanced fatty acid profile, with high levels of oleic and linoleic acids, makes it a good emollient that improves skin softness and smoothness [54]. Almond oil’s primary therapeutic driver is its high α-tocopherol content, a potent antioxidant that protects cell membranes from free radical attack, this property explains its use in photoprotection, mitigating UV-induced damage in animal models [51]. Recent clinical trials substantiate its anti-aging potential, showing that daily oral consumption significantly reduces wrinkle severity in postmenopausal women and suggesting a translatable topical effect [55]. Two limitations exist: its higher PUFA content (Cox index 3.12) can cause chemical instability, necessitating added antioxidants, and its allergenicity risk as a tree nut derivative restricts its use in hypoallergenic products and requires labeling. However, oleogels produced from this oil will be rich in essential fatty acids, making them suitable for anti-aging cosmetics and wound healing products.

The composition of polyunsaturated fatty acids (PUFAs) indicates an oil’s capacity to modulate cutaneous inflammatory responses. An oil’s inflammatory profile is determined by its ratio of omega-6 to omega-3 fatty acids, as metabolites from these families often have opposing effects. A high omega-6/omega-3 ratio is generally pro-inflammatory, while a lower, more balanced ratio is associated with anti-inflammatory effects [56]. The two oils show a profound difference in this regard. Almond oil contains significant linoleic acid (omega-6; 23.772 g/100 g) but lacks detectable alpha-linolenic acid (ALA; omega-3), resulting in a highly skewed, pro-inflammatory profile. In contrast, camellia oil contains both linoleic acid (8.503 g/100 g) and a measurable amount of ALA (0.194 g/100 g), yielding an omega-6/omega-3 ratio of approximately 44:1. While this ratio is high, the presence of ALA confers a more balanced and less inflammatory profile compared to almond oil. This distinction is relevant for dermatological applications, as excess omega-6 can exacerbate inflammatory conditions like eczema and psoriasis. Camellia oil’s more balanced profile makes it a theoretically superior choice for formulations designed to soothe inflamed skin. Therefore, camellia oil is the technologically and therapeutically superior choice for stable, high value oleogels intended for advanced and compromised dermatological care. Almond oil remains a functional, cost-effective solvent option for skincare applications in non-allergic consumers.

### 2.2. Textural Analysis

Developing oleogel dressings for skin repair systems requires understanding the relationships between formulation parameters and textural properties. An interplay among gelling agent properties, concentration, and interactions with an oil phase dictates oleogel firmness and consistency. Our results show that while oleogelator concentration is a primary determinant, vegetable oil influence varies significantly, creating distinct behavioral profiles for each of the three agents (Table 2).

**Figure 1:**
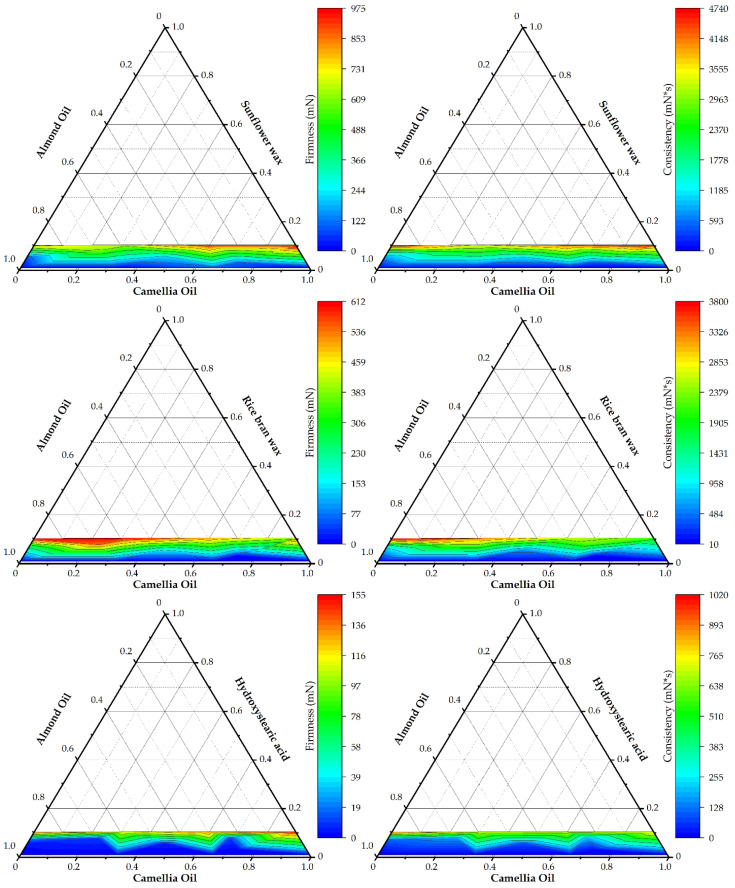
Ternary diagrams for the effect of ingredients on oleogel textural parameters: firmness (**left**) and consistency (**right**). Essential oil blend constant at 0.05 fraction.

SFW was identified as the most potent structuring agent, with its gelling capability showing direct proportionality to its concentration (Figure 1). At high fractions (0.100), SFW produced rigid and cohesive oleogels, achieving highest firmness (up to 971.75 mN) and consistency (up to 4739.36 mN·s). Its potency is strongly supported by the literature, which attributes SFW’s effectiveness to its crystallization into small, needle-like or platelet structures that create a fine, dense, and highly interconnected network [57]. A key SFW attribute was its efficiency at low concentrations; a minimal 0.010 fraction was sufficient to form stable, soft gels (“Sf”) with low firmness (1.14–2.97 mN). Agreement is found with multiple studies that report a minimum gelling concentration (MGC) for SFW as low as 0.5–1.5% in various vegetable oils, confirming it as one of the most effective natural waxes [58,59]. Unlike RBW, SFW performance appeared less dependent on oil type, yielding high firmness and consistency in formulations rich in either camellia or almond oil. Such robustness makes SFW a versatile and predictable structurant for formulation design [60].

RBW also demonstrated potent gelling capabilities, forming oleogels with a firm visual appearance (“Fr”) across nearly all concentrations and reaching a maximum consistency of 3794.18 mN·s. However, its structuring efficiency was notably influenced by the oil phase. A synergistic interaction with almond oil was observed, where high concentrations of almond oil yielded substantially higher firmness and consistency values compared to formulations rich in camellia oil. The observed behavior is consistent with scientific understanding. RBW tends to form larger dendritic or spherulitic crystals which can result in a less homogeneous and comparatively weaker gel than SFW at an equivalent concentration [61]. Oil composition, particularly its triacylglycerol (TAG) profile, can influence oleogelator solubility and crystallization kinetics. An oil that is a poorer solvent for a given wax, as hypothesized for almond oil in the present case, will promote more extensive crystallization and a stronger gel network. RBW’s dependency on oil type has been noted elsewhere; for example, its MGC was found to be much higher in rice bran oil (its native source, in which it is more soluble) than in other vegetable oils [62].

In contrast, HSA was the least potent of the three structuring agents tested. As a low molecular weight organogelator (LMWOG), HSA functions differently from waxes, self-assembling through hydrogen bonding to form a three-dimensional network of long, crystalline fibers [63]. While capable of immobilizing oil, its fibrillar network is generally less dense and mechanically robust than particulate crystal networks formed by waxes. Evidence for its lower potency was clear in the results, as HSA exhibited a distinct concentration threshold for gelation. Below a 0.032 gelling agent fraction, it failed to form a gel network. Such a phenomenon is characteristic of LMWOGs, which require a critical concentration to achieve supersaturation needed for self-assembly [64]. Even at its highest concentration (0.100 fraction), maximum firmness reached only 154.65 mN and consistency was 1019.90 mN·s, approximately 4- to 5-fold lower than oleogels prepared with SFW or RBW.

Analysis of variance (Table 3) confirms that ingredient compositions significantly and predictably affect final oleogel textural properties. For all three gelling agents—sunflower wax (SFW), rice bran wax (RBW), and 12-hydroxystearic acid (HSA)—the overall predictive models for firmness and consistency were statistically significant (*p* < 0.001). Ingredient interaction analysis revealed a critical pattern. For all three gelling agents, interactions between the gelling agent (x_1_) and both vegetable oils—almond oil (x_2_) and camellia oil (x_3_)—were statistically significant (*p* < 0.01). The findings indicate that vegetable oils are not inert solvents but actively modulate the gel network structure. Conversely, the gelling agent–essential oil blend interaction (x_1_*x_4_) was not significant. The gelling agent primarily determines texture, and the vegetable oil type is a significant secondary factor, while the essential oil blend’s interactive effect was negligible. The lack-of-fit tests were non-significant for all models (*p* > 0.05), confirming adequate representation of the experimental space without systematic bias.

The fatty acid composition analysis (Table 1) revealed significant compositional differences between the carrier oils that directly influence oleogel performance. Camellia oil demonstrated superior gelling compatibility compared to almond oil, a result attributed to its distinct lipid profile, which is characterized by a higher monounsaturated fatty acid (MUFA) content (79.4% vs. 66.8%) and a substantially lower polyunsaturated fatty acid (PUFA) content (8.7% vs. 23.8%). This compositional difference was reflected in the experimental results, where formulations with high camellia oil content (>85%) consistently produced firmer gels, while almond oil-dominant formulations (>65%) resulted in substantially reduced firmness.

**Figure 2:**
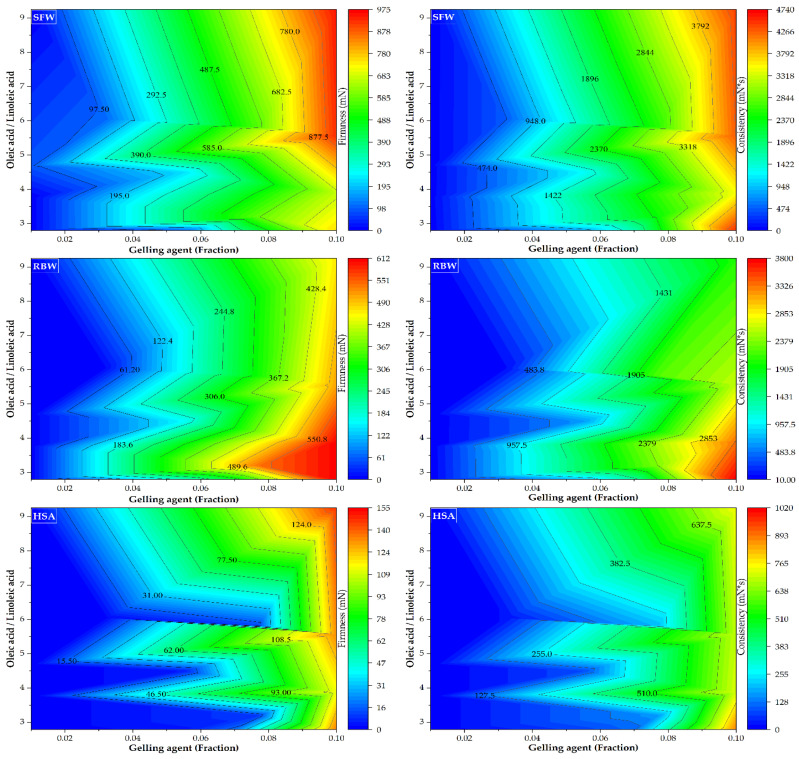
Effects of oleic/linoleic fatty acid ratio and gelling agent concentration on firmness and consistency.

The oleic-to-linoleic acid ratio (Figure 2) emerged as a critical parameter in dictating gel structure, with camellia oil exhibiting a 232% higher ratio (9.26 vs. 2.79) than almond oil. The uniform molecular geometry of oleic acid, primary component of camellia oil, minimizes steric hindrance and acts as a highly compatible solvent phase that facilitates organized gelling agent crystallization. Conversely, the multiple cis-double bonds in linoleic acid (abundant in almond oil) introduce spatial disorder, disrupting intermolecular alignment and yielding structurally weaker networks. This is a fundamental difference that impacts crystallization; as the predominant oleic acid in camellia oil facilitates more organized molecular arrangements that are conducive to the crystallization processes of the gelling agents, the single cis-double bond in oleic acid creates a defined angular bend (“kink”) in the acyl chain [65]. This configuration, while preventing dense packing characteristics of saturated fatty acids, yields a uniform molecular shape. In the oleogel system, oleic acid molecules arrange orderly around the crystallizing oleogelator (e.g., wax esters). Arrangements like these minimize disruption to gelling agent self-assembly and crystallization, acting as a compatible solvent phase.

In camellia oil medium, oleic acid’s compatible nature facilitates more efficient gelling agent crystal nucleation and growth. A process that creates a denser, more homogeneous network of small, well-defined crystals, such a microstructure effectively immobilizes the liquid oil phase via capillary forces, yielding superior gel strength, firmness, and oil-binding capacity, because stable β-polymorphic crystals, characteristic of waxes, likely form optimally in this environment [66]. These molecular interactions directly affect crystallization kinetics and resultant crystal network morphology, which collectively dictate the oleogel’s macroscopic textural properties.

In contrast, a high concentration of linoleic acid in almond oil introduces molecular disorder, which disrupts the efficiency of crystal lattice formation and results in weaker gel networks. Two cis-double bonds in linoleic acid create a more complex, contorted three-dimensional structure; an irregular geometry generates significant steric hindrance, which is a physical impediment disrupting the intermolecular alignment required to form an efficient crystal lattice [66]. This mechanism explains the superior performance of camellia oil-based systems. A higher linoleic acid concentration, as in almond oil, introduces a crystallization antagonizing structuring agent. Crystallization kinetics are likely impeded and the resulting crystal morphology altered, favoring larger, more irregular, or dendritic crystalline structures; a network of such crystals functions less efficiently, possessing fewer junction zones and a more heterogeneous pore size distribution [67]. Consequently, this inefficiency yields a mechanically weaker structure, manifesting as significantly reduced gel firmness. The structuring agents themselves, such as SFW with its stable β-polymorphic crystals and RBW with its good oil compatibility, provide the foundational network that is either supported or hindered by the fatty acid composition of the surrounding oil phase.

In brief, camellia oil’s superior gelling performance results directly from its high oleic acid and low linoleic acid concentrations. Oleic acid’s molecular geometry fosters an environment suitable for efficient gelling agent crystallization, forming a robust, firm structural network. In contrast, steric hindrance from a higher linoleic acid concentration in almond oil disrupts crystallization, resulting in a demonstrably weaker gel. Therefore, fatty acid composition, and specifically the O/L ratio, is a critical parameter for predicting and controlling oleogel textural properties. These findings significantly impact rational oleogel design for dermal care product development requiring precise textural attributes.

As shown in Table 4, the sunflower wax (SFW) model exhibits a large, positive linear coefficient for the gelling agent (x_1_), contrasted by significant negative interaction terms with incorporated oils. This structure indicates that oil dilution attenuates SFW gelling efficiency. The strong negative interaction with camellia oil suggests competitive molecular interactions become prominent at higher oil concentrations. Conversely, the rice bran wax (RBW) model indicates enhanced synergy with the oil phase, reflected in a more balanced coefficient pattern and robust R^2^ values. The negative oil coefficients are proportionally smaller than for SFW, indicating superior oil compatibility and diminished dilution effects. The 12-hydroxystearic acid (HSA) models exhibit distinct behavior, characterized by positive coefficients for almond oil (x_2_) and camellia oil (x_3_). This suggests that, unlike wax-based systems, these oils may facilitate network formation, potentially via co-crystallization mechanisms with the gelling agent. HSA’s overall gelling capacity, however, was limited.

The mathematical models developed elucidated fundamental structure–property relationships for three gelling agents in oleogel systems. High coefficients of determination (R^2^), ranging from 0.913 to 0.983, demonstrate model robustness, accurately predicting firmness and consistency from ingredient mass fractions. This predictive capability enables formulation reverse-engineering for specific textural targets, streamlining product development by minimizing empirical laboratory work, thereby reducing costs and time.

### 2.3. Oleogel Optimization

Balanced textural properties are required for oleogel dressing formulation to enhance stability, prevent disintegration, and ensure a favorable sensory profile. Excessively firm oleogels are uncomfortable to apply, whereas overly soft ones lack stability and efficacy. Developed oleogels were optimized by referencing the commercial product mechanical properties firmness (mN) and consistency (mN*s). The objective optimization (Equation (3)) was used to find three oleogel formulations (one for each gelling agent) that come close to the reference gel (Table 5). Response variables were weighted on a 1-to-5 importance scale: firmness (5), consistency (4). Essential oil blend fraction (0.050) was kept fixed to achieve the maximum therapeutic efficiency provided by these bioactive compounds and considering that no significant interaction occurred between x_4_ and other components (Table 4).

Oleogels optimization yielded three formulations (one for each oleogelator) with textural properties statistically indistinguishable from the commercial reference product. The optimization model produced high desirability scores for all variants, with the RBW formulation achieving the highest value (D = 0.986). Validation experiments confirmed that the oleogels’ mechanical properties aligned with the control. The firmness of SFW, RBW and HSA formulations approximated the control’s 5.117 mN value. Likewise, the consistency of all three formulations matched the 80.385 mN*s reference. The Dunnett test verified this textural alignment by comparing each formulation against the control. All *p*-values for firmness and consistency exceeded the α = 0.05 significance threshold. The statistically insignificant differences confirm that the developed models produced three oleogels with textural profiles equivalent to target benchmark. Further analytical and functional testing was performed exclusively on the texturally optimized gels.

### 2.4. Differential Scanning Calorimetry

**Figure 3:**
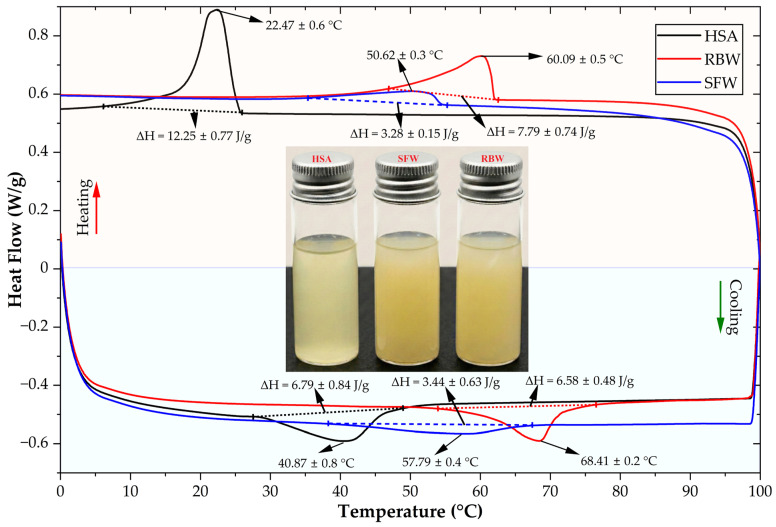
Thermograms comparison of optimized oleogels formulations. SFW: Sunflower wax; RBW: Rice bran wax; HSA: 12-hydroxystearic acid.

Differential Scanning Calorimetry (DSC) quantifies energy transitions during oleogelator network formation and melting to characterize oleogel thermal behavior. The resulting thermograms (Figure 3) and thermal parameters (Table 6) reveal a distinct thermal stability hierarchy modulated by the carrier oils’ fatty acid composition. This relationship provides a basis for understanding the structure–function correlations governing system performance in dermal applications. Rice bran wax (RBW) oleogel formulated predominantly with camellia oil (66%) exhibited superior thermal stability. Upon heating, oleogel melted over a narrow thermal event range of 15.25 °C, tightest among all three systems, with onset (Ts), peak (Tp), and end (Te) temperatures at 46.99 ± 0.73 °C, 60.09 ± 0.5 °C, and 62.24 ± 0.68 °C, respectively. A narrow melting range suggests uniform, highly ordered crystalline lattice formation, where components melt cohesively [68]. Such behavior is likely promoted by a camellia oil base with high oleic acid content (78.75%) and correlates with elevated firmness observed in textural analysis [61]. Upon cooling, crystallization began at Ts = 76.53 ± 0.52 °C, also occurring over a relatively narrow range (22.60 °C), indicating consistent, rapid network formation from melt. Such thermal robustness, supported by its stable oil base (Cox index: 1.71), makes RBW oleogel a candidate for heat-resistant formulations requiring a strong barrier function, such as solid balms or protective ointments. However, a key formulation challenge arises from a trade-off between stability and sensory perception, as properties that make RBW a strong structurant can impart an undesirable heavy or waxy skin feel.

Sunflower wax (SFW) oleogel, formulated with most of the almond oil (55.4%), demonstrated a lower, yet still robust, thermal profile. Melting initiated at Ts = 35.36 °C and concluded at Te = 55.26 °C, spanning a broad 19.90 °C thermal event range. A wider range, compared to RBW, suggests a more heterogeneous crystalline network with varied crystal sizes, potentially influenced by almond oil’s higher linoleic acid content (23.77%), which can introduce molecular disorder [59]. Such heterogeneity is even more pronounced during network formation, as its cooling thermogram shows crystallization occurring over the widest range among all oleogels (29.25 °C), indicating a complex, multi-stage process. Melting enthalpy was significantly lower than RBW, at 3.28 ± 0.15 J/g. SFW structuring efficiency, attributed to its unique crystallization into fine, needle-like structures forming a dense network at low concentrations, offsets this [58]. For dermal applications, such balance is highly advantageous. Combined with almond oil’s emollient properties, SFW provides necessary product stability while creating a desirable velvety, non-greasy skin feel, making it useful for cosmetic creams and lotions. Furthermore, as a film-former, it can create a semi-occlusive barrier on skin, reducing transepidermal water loss and acting as a protective emollient.

In contrast, hydroxystearic acid (HSA) oleogel, formulated primarily with almond oil (68%), displayed a fundamentally different thermal profile. Melting began at a very low onset temperature (Ts) at just 6.08 °C and was completed by 25.93 °C. Low-temperature melting over a broad 19.85 °C range indicates a network only stable at or below room temperature. However, HSA’s system recorded the highest melting enthalpy among all gels (12.25 ± 0.77 J/g); a seemingly contradictory result is characteristic for low molecular weight organogelators (LMWOGs) like HSA, because unlike waxes, HSA self-assembles into long, crystalline fiber networks held together by strong, specific hydrogen bonds [69]. While the HSA formulation exhibited a melting peak (Tp) near room temperature (22.47 ± 0.6 °C), this thermal transition should not be interpreted as a complete loss of physical integrity at skin temperature. Rheological amplitude sweeps performed at 25 °C confirmed that the HSA oleogel maintains a solid-like behavior (G′ > G″) with a storage modulus (G′) exceeding 2 × 10^4^ Pa. This discrepancy between thermal and mechanical profiling is characteristic of the fibrillar “self-assembled fibrillar network” (SAFIN) of HSA. Unlike crystalline waxes that melt abruptly, the HSA network exhibits a broad yielding zone and “ductile” behavior, maintaining structural cohesion up to shear stresses significantly higher than the wax-based systems (89.51 Pa vs. 22–28 Pa). Thus, the low Tp facilitates a “melting-upon-application” sensory profile without compromising the dressing’s retention capability on the wound bed.

A high enthalpy reflects the energy needed to break numerous bonds simultaneously, even though its overall structure melts at a low temperature. Interestingly, its crystallization occurred over the narrowest cooling range (21.41 °C), suggesting a highly specific and uniform self-assembly process. While such a mechanism results in a mechanically weaker gel, it is highly valuable for advanced dermal therapies. Its fibrous network serves as a rate-limiting matrix for controlled and sustained active ingredient release, a critical function for applications like wound management [70]. Furthermore, formulation components are intrinsically beneficial for treating compromised skin conditions like eczema and atopic dermatitis; its almond oil base is rich in skin barrier-essential linoleic acid while HSA itself, as a fatty acid, is a natural skin lipid barrier component and can provide anti-inflammatory and moisturizing properties [46]. Such a combination is ideal where both barrier repair and controlled delivery of actives are needed.

The DSC results clearly delineate a thermal stability order for developed oleogels, ranked as RBW > SFW > HSA. This order corresponds directly to divergent functional applications in dermal care. RBW’s high melting point and enthalpy correlate with a robust, highly crystalline structure, ideal for creating heat-stable products where structural integrity is a primary concern. SFW provides balance between thermal stability and gelling efficiency, making it a versatile structurant for cosmetic formulations where pleasant sensory profiles are crucial. Finally, HSA operates via a distinct hydrogen bonding mechanism; its thermally sensitive, fibrous network is less suited for structural applications but presents advantages as a platform for therapeutic systems that require controlled drug delivery and inherent biocompatibility for skin healing.

### 2.5. Thermogravimetric Analysis

**Figure 4:**
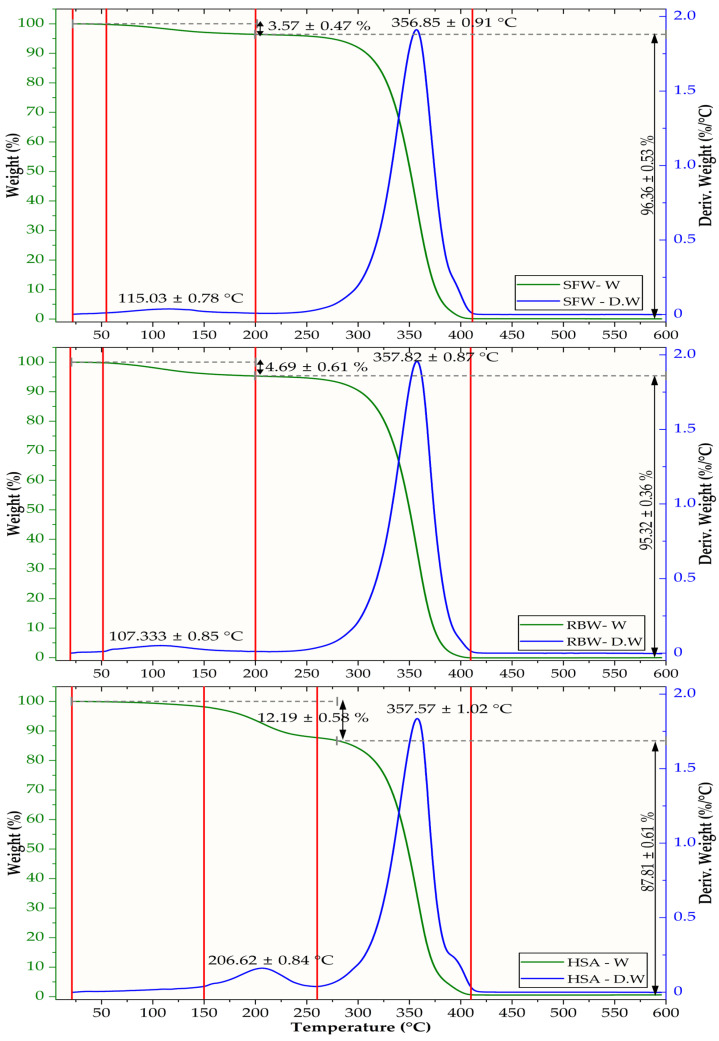
Thermogravimetric profiles of optimized oleogels formulations. SFW: Sunflower wax; RBW: Rice bran wax; HSA: 12-hydroxystearic acid.

Non-covalent forces—including van der Waals interactions, dipole–dipole interactions, and hydrogen bonding (H-bonding)—dictate oleogel functionality and structural integrity [71]. Efficacy and commercial applicability require multi-scale characterization of oleogels, from molecular bonds to macro-scale mechanical properties. Thermogravimetric analysis (Figure 4) provides complementary data on the chemical and thermal degradation stability of the oleogel components and composite system, yielding critical information on processing limits and network integrity.

Formulated with 55.4% almond oil, sunflower wax (SFW) oleogel exhibits a thermal profile characteristic of a stable, homogeneous wax–oil system. The TGA curve indicates a 3.57 ± 0.47% initial mass loss up to approximately 200 °C attributed to the evaporation of volatile essential oil components, followed by a single, sharp thermal degradation event of the lipid matrix that accounts for 96.38 ± 1.55% of the mass. This monophasic degradation, with a 356.88 ± 0.91 °C peak temperature (Tp), confirms the high compatibility and co-decomposition between the SFW long-chain esters and the almond oil triacylglycerols. The Tp value depends primarily on the stability of the oil phase, aligning with reported degradation temperatures for vegetable oils [72]. The highest thermal stability among these three systems was demonstrated by rice bran wax (RBW) oleogel (66.0% camellia oil). Following a 4.69 ± 0.61% initial mass loss from its essential oils, its primary degradation event (95.32 ± 1.36% mass loss) occurs at 357.82 ± 0.87 °C (Tₚ). This value is virtually identical to that of the SFW system, as the global thermal resistance is governed by the triglycerides of the carrier oils, which possess comparable volatilization points. Given the low concentration of gelling agents (<10%), the “lipid matrix effect” dominates the behavior of thermal degradation.

In contrast to wax-based systems, 12-hydroxystearic acid (HSA) oleogel exhibits the most complex and least stable thermal profile. Its TGA curve shows two distinct degradation stages. A first, significant mass loss (12.19 ± 0.38%) begins at a higher onset temperature and peaks at 206.62 °C. This loss is substantially greater than a ~5% contribution from essential oils, indicating that it also involves initial decomposition of the HSA gelling agent itself. This behavior is consistent with the structure of HSA, a hydroxylated fatty acid, which may undergo dehydration or decarboxylation at lower temperatures compared to stable esters found in natural waxes [73]. Its main degradation event (87.81 ± 0.61% mass loss) occurs at 357.57 ± 1.02 °C. Although this Tₚ is numerically similar to other oleogels, its overall profile indicates lower stability due to an earlier onset of significant degradation. This degradation pattern is a consequence of contributions from both its gelling agent and its oil phase (68.0% almond oil). Although its main degradation event peaks at 357.57 ± 1.02 °C (like the other systems), the overall profile indicates lower thermal resilience due to the earlier onset of significant network degradation.

A physicochemical comparison reveals that each oleogel’s thermal degradation directly results from its components’ molecular structure. RBW’s superior stability derives from the synergy between the robust, high-melting-point esters in rice bran wax and the high oleic acid content of camellia oil. Oils rich in monounsaturated fatty acids (MUFAs), such as oleic acid, possess higher thermal decomposition temperatures than those rich in polyunsaturated fatty acids (PUFAs) because a single double bond is less susceptible to thermal cleavage than multiple double bonds [74]. In contrast, SFW oleogel’s stability is compromised by its almond oil base, which has a significant PUFA content (23.8%). Although a single, sharp degradation peak suggests a homogenous system, the less stable oil phase makes this oleogel more susceptible to thermal stress than its RBW counterpart, explaining its intermediate stability. HSA oleogel presents a unique case where the gelling agent itself introduces thermal weakness. Its two-stage degradation is characteristic of functionalized fatty acids. Initial mass loss around 207 °C results from hydroxyl and carboxyl group decomposition, likely via dehydration and decarboxylation reactions that require less energy than C-C bond cleavage in the hydrocarbon backbone [75]. Following this initial breakdown, the remaining HSA hydrocarbon structure and almond oil degrade at a higher temperature (~358 °C). This biphasic degradation fundamentally limits the processing window for HSA-based systems.

The comparative analysis reveals that while the wax-based oleogels (SFW and RBW) maintain structural integrity until temperatures exceed 300 °C, the HSA system presents a more limited thermal processing window. These findings have significant implications for dermocosmetic formulation. The robust stability of the wax–camellia oil systems makes them ideal for products undergoing high-temperature manufacturing, such as the hot-filling of balms and sticks. In contrast, the thermally sensitive HSA system is better suited for specialized, cold-processed applications, serving as a vehicle for heat-labile actives where preserving bioactivity outweighs thermal resilience. Ultimately, TGA profiles provide a critical guide for selecting an oleogel system that aligns with manufacturing constraints and final application requirements.

### 2.6. Fourier Transform Infrared (FTIR) Spectroscopy

The analysis of complex natural products, such as essential oils, demands analytical techniques that are both rapid and information-rich. Fourier Transform Infrared (FTIR) spectroscopy has emerged as a cornerstone technology in this domain, offering profound insights into the chemical architecture of these intricate mixtures, and its power lies in its ability to generate a unique “molecular fingerprint” based on the vibrational behavior of chemical bonds.

**Figure 5:**
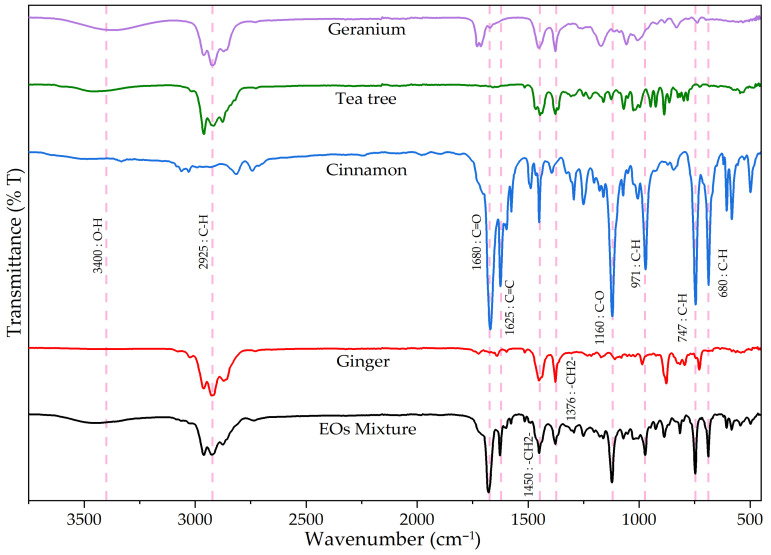
FTIR spectra of individual essential oils and their optimized mixture.

Figure 5 depicts the spectrum for essential ginger oil, primarily composed of zingiberene. The observed bands signified a typical hydrocarbon structure. Specifically, C-H stretching vibrations from alkyl (CH2 and CH3) bonds were detected at 2925.15 cm^−1^ and 2961.66 cm^−1^, with an additional methyl group (-CH3) C-H stretch at 2871.35 cm^−1^. C=C stretching in alkenes at 1640.04 cm^−1^ implied sesquiterpene presence. Further C-H bending vibrations, typical for long aliphatic chains (methyl and methylene groups), were seen at 1450.29 cm^−1^ and 1376.96 cm^−1^. C-O or C-O-C stretching vibrations at 1170.50 cm^−1^ and 1109.75 cm^−1^ pointed to possible oxygenated compounds or ethers. Finally, signals at 986.17 cm^−1^, 728.93 cm^−1^, 877.35 cm^−1^, and 541.30 cm^−1^ all related to out-of-plane C-H bending, common in unsaturated or aromatic compounds. This spectrum thus revealed that ginger EO was a complex mixture. It comprised sesquiterpenes, identified by C=C bond vibration around 1640 cm^−1^, and oxygenated monoterpenes, suggested by C-O vibrations in the 1100–1200 cm^−1^ range.

Overall, this spectrum is characteristic of an EO composed primarily of aliphatic hydrocarbons, unsaturated compounds (terpenes), and oxygenated compounds (ethers, alcohols). Strong bands near 2900 cm^−1^ confirmed abundant C-H bonds, while the 1640 cm^−1^ band reinforced C=C double bond presence, typical for terpenes. Prominent C-H and C=C stretching vibrations confirm a composition rich in sesquiterpene hydrocarbons. While these non-polar structures facilitate lipid-rich biological membrane interaction, its potent bioactivity stems largely from minor phenolic constituents, such as gingerols and shogaols [76]. These compounds inhibit key enzymes in the arachidonic acid cascade, reducing pro-inflammatory prostaglandin and leukotriene production [77]. Furthermore, they suppress the master inflammatory transcription factor NF-κB, critical for downregulating the inflammatory healing phase, creating a microenvironment conducive to skin repair [76].

Infrared spectrum bands for tea tree essential oil (Figure 5) revealed the following: 3460.01 cm^−1^ corresponded to hydroxyl (OH) bonds, denoting alcohols or phenols; 2960.75, 2917.04, and 2876.40 cm^−1^ were C-H stretching peaks, typical for methyl (–CH_3_) and methylene (–CH_2_) groups common in hydrocarbons and their derivatives; 1446.56 cm^−1^ is associated with C-H deformation in methyl and methylene groups; 1377.39 cm^−1^ indicated CH_3_ (methyl) deformation from terpenes; 1306.56, 1223.44, 1160.73, and 1125.70 cm^−1^ suggested possible C-O bonds (esters or alcohols); 1069.59 cm^−1^ and 1025.86 cm^−1^ were indicative of C-O bonds, possibly associated with ester structures; 948.24 cm^−1^ matched terpene structure; 925.24, 886.92, 863.93, and 824.77 cm^−1^ are distinctive C-H deformation bands in terpenes or aromatic compounds; 789.97 and 781.92 cm^−1^ related to terpene functional groups and C-H deformation; 544.69 cm^−1^ was an uncommon band, possibly a bond vibration signifying complex groups or less frequent structures.

Signals in the 3400–3500 cm^−1^ region implied OH groups, a feature for EOs with antimicrobial and anti-inflammatory properties. A pronounced, broad O-H stretching band (~3460 cm^−1^) and distinct C-O stretching peak define tea tree oil’s spectroscopic features, reflecting its high terpinen-4-ol (a tertiary alcohol) concentration [78]. This hydroxyl group constitutes the “active site” responsible for the oil’s potent, broad-spectrum antimicrobial activity, crucial for preventing open wound infection. The mechanism involves the lipophilic terpene backbone inserting into the bacterial cytoplasmic membrane, while the polar hydroxyl group disrupts membrane integrity, a process that causes essential intracellular component leakage and cell death [79]. Beyond its antimicrobial role, terpinen-4-ol is an immunomodulator, suppressing pro-inflammatory cytokines production, helping resolve inflammation and facilitating transition to the proliferative healing phase [80].

Main bands in the cinnamon essential oil spectrum were observed at 3029.01 cm^−1^ and 2914.83 cm^−1^, linked to hydrocarbon C-H bond stretching. This signified methyl (–CH_3_) or methylene (–CH_2_) groups common to aliphatic structures and aromatic rings bearing aliphatic substituents. Carbonyl group (C=O) stretching vibrations, generally associated with aldehydes and ketones, were present at 1670.80 cm^−1^. In cinnamon, this matched its main component, cinnamaldehyde. The aromatic structure was further detailed by several bands. A band at 1624.30 cm^−1^ represented aromatic C=C bonds, attributed to cinnamaldehyde. C-H bond deformations in aromatic rings were visible at 1450.02 cm^−1^, 1575.59 cm^−1^, and 1488.29 cm^−1^. Aromatic presence was also verified by out-of-plane C-H bond deformations at 971.01 cm^−1^, 844.41 cm^−1^, and 687.16 cm^−1^, and by out-of-plane ring deformations at 605.65 cm^−1^ and 582.20 cm^−1^. Distinctive alcohols and ethers are denoted by C-O bond vibrations at 1294.13 cm^−1^ and 1250.26 cm^−1^. Further C-O stretches, also associated with ethers or alcohols, were detected at 1160.73 cm^−1^ and 1120.79 cm^−1^; such C-O bonds are typical in phenolic compounds or ethers within essential oils.

Cinnamon oil’s FTIR spectrum is dominated by signals from its primary component, cinnamaldehyde, whose functional groups are directly linked to its potent therapeutic skin repair properties [81]. An intense carbonyl (C=O) peak (~1670 cm^−1^) and aromatic C=C stretching band (~1624 cm^−1^) arise from the α,β-unsaturated aldehyde structure. This motif is highly antimicrobial, irreversibly inactivating essential bacterial proteins and disrupting cell membrane integrity, preventing wound colonization and biofilm formation [82]. The same molecule exerts powerful anti-inflammatory effects by suppressing major signaling pathways, reducing inflammation and supporting the healing cascade [83].

Geranium essential oil was composed mainly of alcohols like geraniol. The IR spectrum (Figure 5) presented a band at 3370.44 cm^−1^ matching O-H bond stretching, typical for alcohols or phenolic compounds. C-H bond stretching (methyl –CH_3_ and methylene –CH_2_) was seen at 2961.16 cm^−1^ and 2924.42 cm^−1^. Further C-H deformation bands (methyl/methylene groups), distinctive of terpenes, were observed at 1452.20 cm^−1^ and 1377.08 cm^−1^. Out-of-plane C-H deformation was noted at 1007.50 cm^−1^. A band at 1712.91 cm^−1^ is associated with carbonyl (C=O) stretching, signifying aldehydes, ketones, or esters; in geranium, these esters possibly included citronellyl acetate. C-O bond stretching at 1170.26 cm^−1^ and 1057.14 cm^−1^ implied other alcohols, esters, or ethers, such as geranyl acetate. Additional signals at 831.66 cm^−1^ and 737.95 cm^−1^ related to out-of-plane C-H deformation (aromatic rings or alkenes) from terpenes or similar components. An uncommon band at 532.53 cm^−1^ possibly corresponded to deformation vibrations in complex, less frequent structures. Overall, the spectrum indicated that geranium essential oil comprised chiefly alcohols (geraniol), esters (citronellyl), and various terpene-type compounds.

Geranium oil’s key functional groups, evidenced by a strong, broad O-H stretch (~3370 cm^−1^) and a C=O stretch (~1712 cm^−1^), correspond to its primary constituents: monoterpene alcohols (citronellol, geraniol) and their esters [84]. These groups are central to their multifunctional skin healing role. Hydroxyl groups are crucial for the oil’s antimicrobial activity, disrupting bacterial membranes in a manner like terpinen-4-ol [79]. Furthermore, these components are potent anti-inflammatory agents, reducing edema and inflammatory cell infiltration by modulating NF-κB and MAPK signaling pathways [85]. Beyond defense and immunomodulation, geranium oil actively promotes the proliferative healing phase, because it also increases collagen deposition and stimulates skin cell regeneration, making it a comprehensive therapeutic wound management agent [86].

The FTIR spectrum for the essential oil (EO) mixture (39.1% cinnamon, 31.7% ginger, 14.7% geranium, 14.5% tea tree) provided a composite spectroscopic signature confirming key functional group presence from each constituent oil. Relative absorption band intensities in the mixture’s spectrum were consistent with each oil’s weighted contribution. As cinnamon oil was the most abundant component, its characteristic spectral features were most prominent. A very strong, sharp absorption band observed at approximately 1680 cm^−1^ was unequivocally attributed to carbonyl (C=O) stretching from an aromatic aldehyde, cinnamaldehyde’s defining feature. Furthermore, a sharp peak at ~1625 cm^−1^ (aromatic C=C bond stretching) and distinct, sharp peaks in the low-wavenumber region (<1000 cm^−1^), such as at ~971 cm^−1^ and ~680 cm^−1^, were characteristic of C-H out-of-plane bending in the cinnamaldehyde aromatic ring. An intense, sharp set of peaks was observed in the 2850–3000 cm^−1^ region, centered around 2925 cm^−1^. These bands corresponded to aliphatic C-H stretching vibrations from methyl (–CH_3_) and methylene (–CH_2_) groups. This feature represented a cumulative signal from all four oils, as terpenes and terpenoids formed their structural backbone. High concentrations of both ginger and cinnamon oils, rich in such structures, accounted for these bands’ high intensity. Similarly, distinct C-H bending vibration peaks at ~1450 cm^−1^ and ~1376 cm^−1^ were strong and well-defined, reflecting their presence across all components. A broad absorption band was visible in the ~3400 cm^−1^ region, characteristic of hydroxyl (O-H) group stretching. This band confirmed alcohol and phenol presence, primary constituents of geranium oil (geraniol) and tea tree oil (terpinen-4-ol). This band’s moderate intensity was consistent with the lower relative percentages of these two oils. Oxygenated compound presence was further substantiated by a complex series of overlapping peaks in the 1200–1000 cm^−1^ range, corresponding to C-O stretching vibrations from alcohols and esters.

Combining the four essential oils into a single mixture creates a multi-target therapeutic agent with significant wound healing potential, likely exceeding individual oil efficacy through synergistic interactions. This formulation addresses multifaceted wound pathology by simultaneously combating microbial invasion, modulating inflammation, and promoting skin recovery. The mixture derives its potent antimicrobial profile from combining distinct component mechanisms: aldehyde-driven protein inactivation from cinnamaldehyde [82] and membrane disruption by terpene alcohols in tea tree and geranium oils [78,79]. Furthermore, the blend offers comprehensive anti-inflammatory action, combining COX/LOX inhibition from ginger’s phenolic compounds [77] with broad suppression of NF-κB and MAPK signaling pathways by components from cinnamon, tea tree, and geranium oils [80,85]. Targeting multiple inflammatory cascades allows the mixture to resolve prolonged inflammation that often stalls healing. Finally, this optimized anti-inflammatory and antimicrobial environment supports geranium oil’s tissue-regenerative properties, which actively promotes collagen deposition [86].

**Figure 6:**
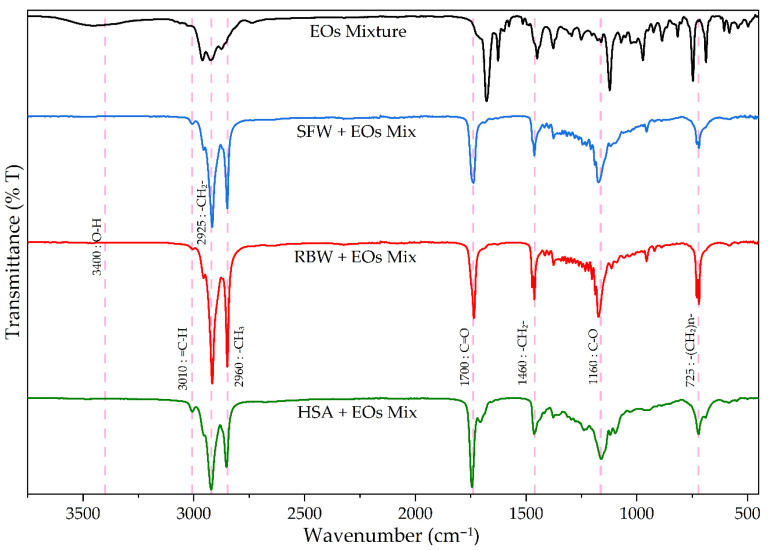
FTIR profiles of optimized oleogels formulations. SFW: Sunflower wax; RBW: Rice bran wax; HSA: 12-hydroxystearic acid.

Figure 6 compares FTIR spectra for the neat essential oil (EO) mixture and three optimized oleogels (SFW, RBW, HSA). The analysis confirms the successful EO incorporation into the oleogel matrix and identifies primary chemical functionalities in final formulations.

Visual inspection reveals that three oleogel spectra are dominated by a different peak set than the pure EOs mixture. While the essential oil blend (black line) exhibits a complex fingerprint region (1500–500 cm^−1^) and characteristic peaks for aromatics, aldehydes, and alcohols, oleogel spectra (blue, red, green lines) are substantially simpler, defined by key vehicle absorptions. Most intense signals in all three oleogel spectra originate from carrier oils (almond, camellia), which constitute the formulation bulk (88–94%, as per Table 6). This dominance is evident in key regions. The C-H stretching region (3100–2800 cm^−1^) contains the most intense bands; oleogel spectra were dominated by a strong, sharp doublet, the hallmark of long fatty acid chains from carrier oil triacylglycerols. This doublet includes asymmetric C-H stretching from methyl (-CH_3_) groups (~2960 cm^−1^) and methylene (-CH_2_) groups (~2925 cm^−1^). A smaller, adjacent shoulder (~3010 cm^−1^) was also present, characteristic of =C-H stretching from unsaturated fatty acids (e.g., oleic, linoleic acid). Carrier oils overwhelmingly contribute to this entire band set.

A second defining characteristic was the strong, sharp carbonyl (C=O) stretch (~1700–1745 cm^−1^), a C=O functional group stretching vibration that defines the oleogels. Its origin is twofold. In SFW and RBW gels, the primary contribution stems from the ester linkages (R-C(=O)-O-R’) of the triacylglycerols (vegetable oils) and long-chain esters (wax gelators), absorbing strongly around ~1745 cm^−1^. In the HSA oleogel (green line), however, this peak centers closer to ~1700 cm^−1^, which is characteristic of the 12-hydroxystearic acid gelling agent’s carboxylic acid C=O group. Finally, this C=O stretch is complemented by a strong band at ~1160 cm^−1^, corresponding to the C-O single bond stretching vibration, another key feature of the ester groups present in the oils and wax gelators [87]. The fingerprint region (1500–500 cm^−1^) confirms carrier oil dominance. Strong peaks are observed at ~1460 cm^−1^ (C-H bending, -CH_2_- groups), ~1160 cm^−1^ (C-O single bond stretch, ester groups), and ~725 cm^−1^ (C-H rocking deformation, long -(CH_2_)n- chains). These signals are classic triacylglycerol structure identifiers. Figure 6 spectra clearly demonstrate that the final oleogel spectroscopic signature is dominated by the vehicle (carrier oils, gelling agents), as expected given that these components account for 95% of total formulation.

Characteristic therapeutic essential oil blend peaks are “masked” or “obscured” by overwhelming signals from the oleogel base. This masking is evident when comparing the EOs mixture spectrum (Figure 6) with its constituent oil spectra (Figure 5). For instance, the most prominent, diagnostic peak—the sharp C=O aldehyde stretch from cinnamaldehyde at ~1680 cm^−1^ (inherited from the pure cinnamon spectrum, Figure 5)—is completely enveloped and obscured by the much stronger, broader C=O absorption from vehicle esters and/or acids (~1700–1745 cm^−1^). Similarly, the broad O-H band at ~3400 cm^−1^, a key therapeutic marker from alcohols in geranium and tea tree oils (Figure 5), reduces to a very slight, broad shoulder in SFW and RBW oleogel spectra. It is visibly retained only in the HSA oleogel spectrum (green line), as the 12-hydroxystearic acid gelling agent (6.7% concentration) also contains a hydroxyl (O-H) group, adding to this signal. Furthermore, the complex essential oil blend “fingerprint” region (1500–500 cm^−1^), containing unique identifiers for ginger sesquiterpenes (e.g., ~1376 cm^−1^) and cinnamon aromatic bends (e.g., ~971 cm^−1^, ~680 cm^−1^), is also completely overshadowed by strong C-O (~1160 cm^−1^) and C-H bending peaks (~1460 cm^−1^, ~725 cm^−1^) from carrier oils. This masking effect does not indicate essential oil degradation or absence; rather, it confirms their successful dissolution and physical incorporation within the lipid matrix. The final spectrum is an additive composite of all components, where the 5% (*w*/*w*) essential oil contribution is spectrally minor compared to the 95% (*w*/*w*) contribution from the C-H and C=O/C-O dense vehicle.

FTIR analysis thus confirms that the final oleogels are stable physical mixtures, with the base vehicle’s chemical identity defining the primary spectroscopic profile. While detailed quantitative EO analysis relies on chromatographic techniques (as performed in the authors’ previous work), FTIR serves as an invaluable, rapid, and complementary method. It provides a holistic “molecular fingerprint” ideal for quality control, allowing for rapid verification of the oleogel base’s structural identity and batch-to-batch consistency.

### 2.7. Rheological Characterization

**Figure 7:**
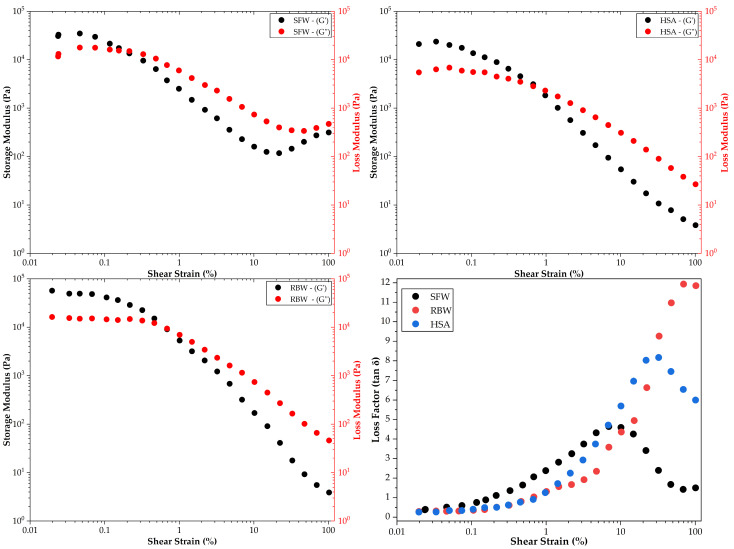
Amplitude sweeps for optimized oleogels formulations. SFW: Sunflower wax; RBW: Rice bran wax; HSA: 12-hydroxystearic acid.

Rheological profiling of oleogel dressings determines their functional performance, governing storage stability, ease of application, and retention at the wound site. While large-deformation texture analysis suggested comparable macroscopic firmness values for the optimized formulations, small-amplitude oscillatory shear (SAOS) measurements exposed profound microstructural differences driven by distinct physicochemical assembly mechanisms. Amplitude sweeps profiles (Figure 7) confirmed that all formulations exhibit solid-like behavior, characterized by a storage modulus (G′) consistently exceeding the loss modulus (G″) within the linear viscoelastic region (LVR). This elastic dominance indicates the formation of a continuous, self-supporting three-dimensional network capable of immobilizing the liquid oil phase; however, the rigidity magnitude varied distinctively among the gelling agents.

Rice bran wax (RBW) oleogel formed the stiffest network, exhibiting a storage modulus (G′) of approximately 56,916 Pa (Table 7)—a rigidity that correlates directly with the thermal data (Table 6), where RBW exhibited the highest melting enthalpy (ΔHm = 7.79 J/g). From a microstructural perspective, this high elastic modulus derives from the inherent crystal habit of rice bran wax esters (C24–C34), which crystallize into high-aspect-ratio, needle-like aggregates. This rheological behavior correlates strongly with the chemical composition of the vehicle; the camellia oil used is rich in monounsaturated fatty acids (MUFAs, ~79% oleic acid). The uniform geometry of oleic acid minimizes steric hindrance, allowing the wax needles to pack densely and “sinter” (fuse) effectively at junction zones [88]. This sintering creates a rigid, permanent-like lattice that resists deformation, explaining the high stiffness values (G′ > 10^4^ Pa). The high concentration of monounsaturated fatty acids (MUFAs) in the camellia oil vehicle (Table 1) likely supports such lattice formation by minimizing steric hindrance during crystal growth, a phenomenon previously identified in wax-based organogels [89]. These stiffness values are consistent with previous studies on rice bran wax oleogels, where G′ values exceeding 10^4^ Pa are commonly reported even at concentrations as low as 5%, confirming its status as a highly efficient structuring agent compared to other natural waxes [90]. Correlating with the textural analysis, this brittle, sintered microstructure explains why RBW formulations rapidly achieved high “firmness” and “consistency” values in the mixture design, acting as a solid balm that requires significant force to fracture.

The sunflower wax (SFW) oleogel exhibited an intermediate stiffness (G′ ≈ 24,350 Pa), approximately 2.3-fold lower than the RBW system. While SFW esters (C_42_–C_60_) typically form strong networks, their performance here is modulated by the almond oil vehicle, chemically characterized by a high content of polyunsaturated linoleic acid (~24%). Physicochemically, the “kinked” geometry of linoleic acid molecules creates steric exclusion zones that disrupt the lateral stacking of SFW crystals, forcing them into a platelet-like morphology rather than interlocking needles; these platelets stack loosely with weaker London dispersion forces at the junctions, creating a network prone to internal sliding [91]. This directly correlates with the textural findings where SFW gels were often classified as “Soft” or required higher concentrations to match the macroscopic firmness of RBW. The “soft glassy” rheology effectively explains the “velvety” sensory profile often attributed to SFW, distinguishing it from the harder, waxy nature of the RBW system.

The 12-hydroxystearic acid (HSA) oleogel displayed the lowest stiffness (G′ ≈ 21,144 Pa), comparable to the SFW system, but driven by a fundamentally different molecular force. Unlike the crystalline waxes, HSA functions as a low molecular weight organogelator (LMWOG) that self-assembles via anisotropic growth into high-aspect-ratio fibrils (SAFIN), stabilized primarily by intermolecular hydrogen bonds between the hydroxyl group at C-12 and the carboxyl headgroup [64]. While this fibrillar architecture creates a pervasive mesh that effectively traps oil, the individual hydrogen bonds are energetically weaker and more flexible than the dense crystalline junctions of waxes, resulting in a lower overall elastic modulus. This chemically distinct mechanism correlates with the textural observation that HSA gels required higher mass fractions to achieve the target firmness, yet they maintained a smooth, cohesive appearance distinct from the crystalline opacity of the wax-based gels.

**Figure 8:**
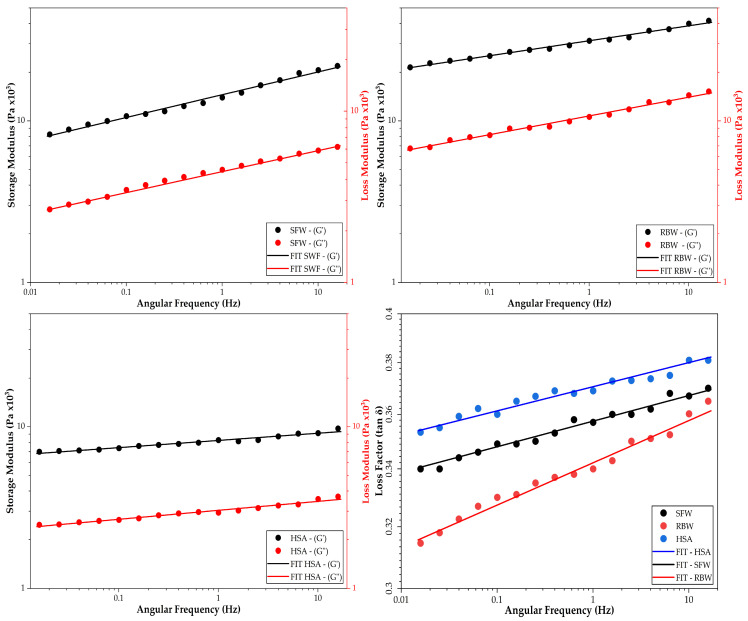
Frequency sweeps for optimized oleogels formulations. SFW: Sunflower wax; RBW: Rice bran wax; HSA: 12-hydroxystearic acid.

Frequency sweep tests (Figure 8) provided a clear insight into the nature of the particle interactions within the gel network. All samples showed a linear increase in G′ with angular frequency, fitting the power-law model (G = K (2π*f*)^n^) with high correlation (R^2^ > 0.98). The frequency index (n), where n = 0 indicates a covalent gel and higher values indicate physical entanglements, revealed distinct stability characteristics (Table 8). HSA formulation exhibited the lowest frequency dependence (n = 0.044), indicating a “strong gel” behavior where the network structure is nearly independent of the timescale of deformation. These findings support the hypothesis that the hydrogen-bonded fibers of HSA form a permanent-like scaffold that resists rearrangement over short timescales [92]. Conversely, SFW oleogel exhibited the highest frequency dependence (n = 0.141), indicative of a “weak gel” or “soft glassy” material. Such behavior suggests that the junction zones between sunflower wax crystals are more transient, potentially allowing for slow structural rearrangements or “creep” over long storage periods, a limitation often cited in wax oleogels with high liquid oil fractions [93]. The observed trend agrees with other works where it was noted that while sunflower wax forms stable gels, they often exhibit higher frequency dependence (n > 0.1) compared to more crystalline waxes like rice bran wax, particularly in unsaturated oil mediums [94].

Beyond the linear viscoelastic region, the yield stress—the minimum force required to initiate flow—is a critical parameter for skin dressings (Table 7). It determines whether the oleogel will remain in place on a vertical wound surface or flow under gravity [95]. RBW demonstrated the highest yield stress (20.91 Pa), consistent with its brittle, sintered network; a yield stress of this magnitude ensures good retention at the application site, preventing the dressing from dripping or migrating onto healthy skin—a crucial factor for maintaining a moist healing environment [96]. Conversely, SFW oleogel exhibited an intermediate yield stress value (14.85 Pa). While beneficial for easy spreading, it poses a functional risk: the material may flow under its own weight if applied in thick layers, potentially compromising the occlusive barrier integrity over time. Such behavior corresponds with the “soft” nature observed in the amplitude sweeps and highlights a trade-off between sensory spreadability and mechanical retention. Similar low yield stress values (<15 Pa) for sunflower wax oleogels in nut oils have been reported before, attributing the low structural resistance to the formation of plate-like crystals that slide past one another more easily than the interlocking needle-like crystals of rice bran wax [97]. Notably, HSA exhibited the lowest yield stress (10.77 Pa), yielding early (Table 7). However, a deeper look at the Flow Point data (where G″ > G′) reveals a unique “ductile” characteristic for HSA. While it yields early, it maintains structural cohesion up to a stress of 89.51 Pa—nearly four times higher than the waxes (SFW: 22.61 Pa; RBW: 28.43 Pa). This broad “yielding zone” suggests that the HSA fibrils, unlike rigid wax crystals, can stretch and align under strain (strain-hardening) before the network catastrophically fails. This ductility allows the dressing to conform comfortably to the wound bed without dripping, offering a significant advantage over the brittle waxes which fracture abruptly.

**Figure 9:**
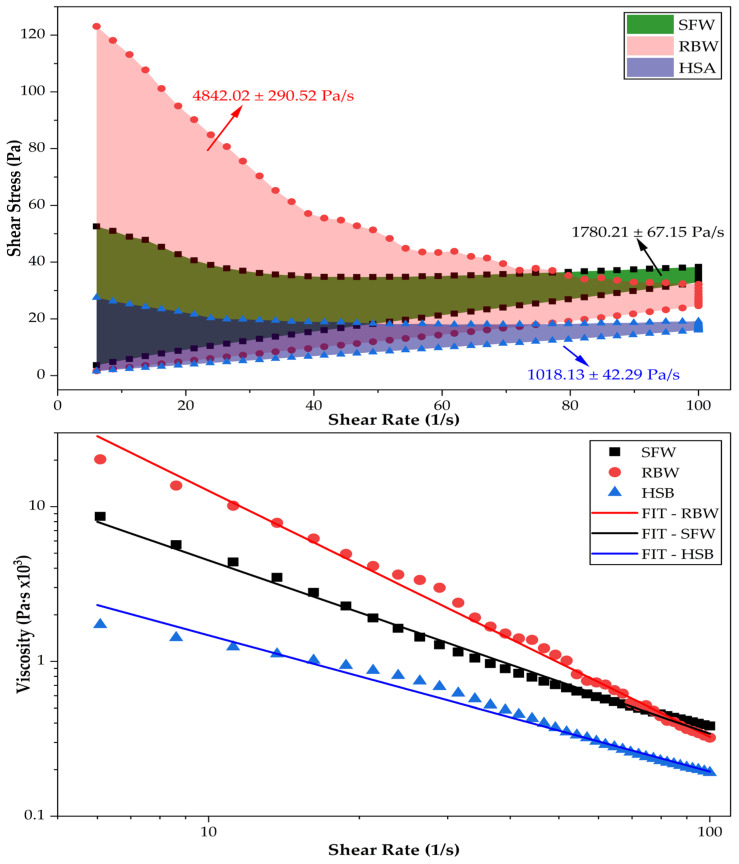
Thixotropic (Pa/s) and viscosity (Pa·s) profiles for optimized oleogels formulations. SFW: Sunflower wax; RBW: Rice bran wax; HSA: 12-hydroxystearic acid. Shaded areas correspond to thixotropy (Pa/s).

Effective skin dressings must exhibit thixotropy; they should shear-thin during application (rubbing) to spread easily and recover their structure rapidly upon cessation of shear to form a protective layer. Viscosity curves confirmed that all oleogels are non-Newtonian, shear-thinning fluids, but their structural recovery paths differed significantly, providing critical insights into the flow mechanics of these systems (Figure 9). Experimental viscosity data exhibited a strong linear relationship on a logarithmic scale, validating the power law model applicability to describe the flow behavior of these oleogels. As illustrated by the solid lines in Figure 9 (bottom), model fits closely track the experimental points across the entire shear rate range (3 to 100 s^−1^). The high coefficients of determination (R^2^), ranging from 0.987 to 0.995 (Table 8), confirm that the pseudoplastic behavior is mathematically consistent and predictable. This good fit indicates that the structural breakdown of the crystal networks occurs uniformly under stress, without artifacts such as wall slip or shear banding that can often complicate the rheological modeling of wax-based systems. The precision of these models allows for reliable prediction of the dressing’s behavior during high-shear processes like pumping or rubbing.

Rice bran wax (RBW) oleogel exhibited the highest consistency index (K ≈ 4.93 × 10^5^ Pa·s), indicating an extremely high resistance to flow at low shear rates, effectively acting as a solid balm at rest. However, it also displayed the most pronounced shear-thinning behavior, evidenced by the steepest slope in Figure 9 and the most negative flow index (n = −1.594). Such a negative slope implies that the RBW oleogel crystalline network undergoes a catastrophic structural collapse when rubbed. The needle-like crystals, initially interlocked in a rigid scaffold, align rapidly under flow, causing the viscosity to plummet by several orders of magnitude. While this dramatic breakdown facilitates spreading, the thixotropic analysis (Figure 9, top panel, shaded area) reveals a great hysteresis loop (4842 Pa/s). A large energy dissipation indicates that the broken network requires significant time and energy to rebuild. Critically, these fracture mechanics suggest that while RBW oleogels provide a robust protective barrier at rest, they may momentarily run off vertical surfaces immediately after application before the network re-sinters [98].

Sunflower wax (SFW) oleogel occupied an intermediate position (K ≈ 1.85 × 10^5^ Pa·s; n = −1.250), with a fit line falling between the RBW and HSA extremes. Its thixotropic area (1780 Pa/s) represents a functional balance, with a network that is sufficiently disrupted by shear to allow for a “velvety” spreadability—smoother than the rigid RBW but with more body than the HSA—while retaining enough structural memory to recover its viscosity efficiently. This intermediate rheological profile makes SFW a versatile candidate for general topical applications where a balance between ease of spreading and post-application retention is required. In sharp contrast, 12-hydroxystearic acid (HSA) oleogel exhibited the lowest consistency index (K ≈ 1.12 × 10^4^ Pa·s) and least aggressive shear-thinning profile (n = −0.881), appearing as the flattest line in Figure 9 (bottom). This profile suggests that the fibrillar HSA network breaks down more gradually and maintains a higher relative viscosity under shear compared to its starting point.

Most critically, the HSA system displayed the smallest thixotropic loop (1018 Pa/s). A minimal hysteresis implies near-instantaneous structural recovery, because hydrogen bonds drive the HSA self-assembly to re-form on a timescale much faster than the nucleation and growth required for wax crystals [99]. The resulting rapid “snap-back” elasticity is highly advantageous for wound care, ensuring that the dressing re-solidifies immediately after the physician stops applying friction; consequently, it prevents dripping and maintains a consistent dosage on the wound bed. Notably, recent work on HSA-based systems corroborates this finding, consistently citing rapid thixotropic recovery as a key advantage for injectable and topical drug delivery systems compared to crystalline wax matrices [64].

### 2.8. Bactericidal Activity

**Figure 10:**
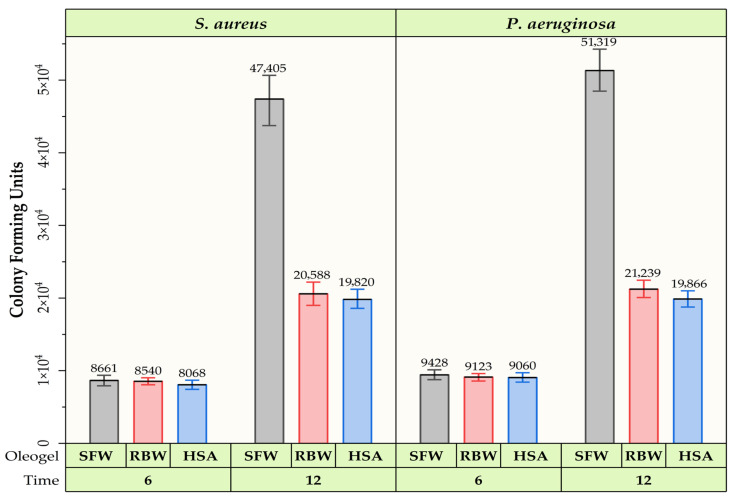
Time-dependent bactericidal kinetics of optimized oleogel formulations against *Staphylococcus aureus* and *Pseudomonas aeruginosa*. Contact time (hours).

Bactericidal assessment (Figure 10) reveals a robust, time-dependent antimicrobial capability of optimized oleogel formulations against both Gram-positive (*Staphylococcus aureus*) and Gram-negative (*Pseudomonas aeruginosa*) pathogens. All three oleogel systems (SFW, RBW, HSA) demonstrated a statistically significant reduction in bacterial viability compared to untreated controls, achieving a >99% reduction (~2 Log_10_ reduction) after 12 h of contact. While oleogels achieved a rapid reduction in S. aureus populations within the first 6 h, kinetic profiles against P. aeruginosa showed a more gradual decline, requiring full 12 h exposure to reach comparable inhibition. Such behavior is consistent with the intrinsic resistance of Gram-negative bacteria, whose outer lipopolysaccharide membrane functions as a selective barrier against hydrophobic agents [100].

Final bactericidal values confirm that these formulations successfully overcome such barriers, validating their potential as broad-spectrum antimicrobial dressings. One-way analysis of variance (ANOVA) statistically validated the bactericidal effects observed at the 12 h endpoint. The results confirmed a highly significant difference in bacterial viability between untreated controls (6.0 Log_10_ CFU/mL) and oleogel treatments (≈4.0 Log_10_ CFU/mL), with an F-value of 1132.00 and a *p*-value < 0.001. Corroborating findings indicate that cinnamon oil-loaded nanoemulsions exhibit superior bactericidal activity against S. aureus compared to *E. coli*—an effect attributed to irreversible cell membrane disruption by cinnamaldehyde [101]. Similarly, studies on tea tree oil formulations show significant log reductions in wound pathogens, suggesting that lipid-based delivery systems enhance the stability and contact time of active terpenes [41]. Observed time-dependent efficacy against P. aeruginosa also aligns with previous work; while essential oils are effective against Gram-negative strains, kinetics remain slower due to limited diffusion rates through outer membrane porins and the action of efflux pumps [102].

The distinct bacterial regrowth observed in the SFW oleogel at the 12 h mark is likely driven by the physicochemical properties of its network. Rheological analysis identified SFW as having the “softest” structure (G′ 2.4 × 10^4^ Pa) with the highest frequency dependence (n = 0.141), indicative of a less dense, more permeable crystal lattice. This porous microstructure likely facilitates a rapid initial “burst release” of the volatile essential oils, leading to their premature depletion at the dressing–skin interface. Once the antimicrobial concentration falls below the inhibitory threshold, the surviving sub-lethally injured bacteria may recover. Additionally, the almond oil vehicle, rich in polyunsaturated fatty acids (23.77%), is more susceptible to oxidative degradation than the camellia oil used in RBW systems; however, further metabolic studies would be required to confirm if bacterial lipases are actively utilizing these lipids as a carbon source during the regrowth phase.

Observed in vitro potency stems primarily from the synergistic phytochemical profile of the loaded essential oil blend (39.1% cinnamon, 31.7% ginger, 14.5% tea tree, 14.7% geranium) releasing from the lipid matrix. FTIR analysis confirms that high concentrations of cinnamaldehyde (carbonyl stretch at ~1680 cm^−1^) and terpinen-4-ol (broad O-H stretch) drive a dual-action mechanism. Functioning as an electronegative electrophile, cinnamaldehyde interferes with electron transfer processes and reacts with nitrogen-containing components (proteins, nucleic acids), effectively inhibiting cell division (FtsZ protein) and ATP generation. Concurrently, lipophilic terpinen-4-ol and geraniol partition into bacterial membranes, disrupting structural integrity and inducing leakage of vital intracellular ions like potassium (K^+^) [82].

While wax-based oleogels (SFW, RBW) rely on crystalline lattices to entrap volatiles, 12-hydroxystearic acid (HSA) systems operate through a distinct self-assembled fibrillar network (SAFIN) driven by intermolecular hydrogen bonding [64]. Such a fibrillar microstructure, characterized by rapid thixotropic recovery, likely facilitates unique diffusion profiles for essential oils, ensuring sustained release without the “burst effect” common in less structured carriers. Furthermore, although current in vitro results measure direct bacterial killing, the medical value for HSA formulations can be potentially superior due to secondary, indirect mechanisms. Consequently, HSA oleogels could offer a dual-therapeutic modality in vivo: direct pathogen eradication via essential oils and simultaneous boosting of innate immunity via the gelator.

The 2-Log bacterial load reduction found indicates an important antimicrobial suppression, essential for wound management. Achieving significant pathogen decrease allows optimized oleogels to mitigate wound sepsis risks and biofilm establishment. Lipophilic environment activity offers specific advantages; unlike rapidly dissolving aqueous hydrogels, oleogel systems provide persistent, hydrophobic barriers protecting wound beds against external contamination while treating existing infections.

### 2.9. Cell Viability and Proliferation

**Figure 11:**
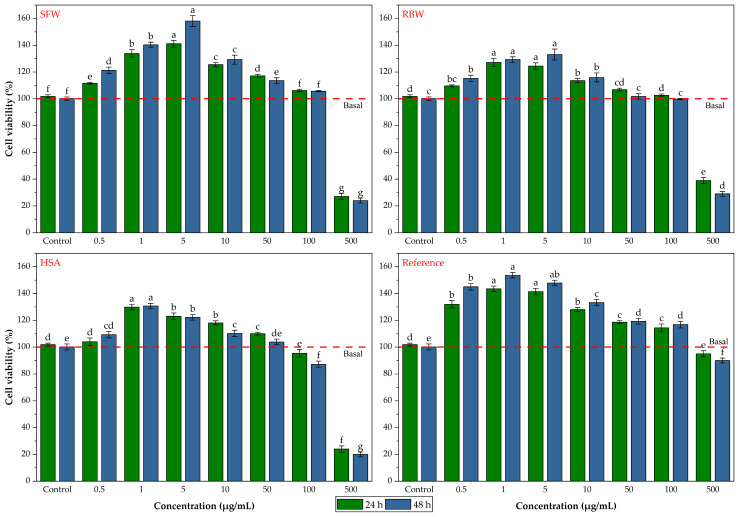
Dose-dependent viability profile of HaCaT keratinocytes exposed to optimized oleogels formulations (SFW, RBW, HSA) over 24 and 48 h. Data are expressed as mean ± SD (n = 3). Different letters: significant difference between treatments (*p* < 0.05). Concentration (μg/mL): essential oil blend equivalent.

The interaction between optimized oleogels and human keratinocytes (HaCaT) revealed a concentration-dependent biphasic response, characterized by a hormetic transition from proliferation to cytotoxicity (Figure 11). At low essential oil blend equivalent concentrations (1–5 μg/mL), all formulations demonstrated high biocompatibility, with cell viability consistently exceeding 100% after 24 and 48 h. Notably, the 5 μg/mL treatment induced the most statistically significant proliferation (*p* < 0.05) compared to the untreated control. Consequently, the 1–5 μg/mL range is identified as the optimal therapeutic window, outperforming higher doses.

Regenerative effects were formulation-dependent; at 48 h, SFW oleogel demonstrated the highest regenerative potency among all formulations, achieving a peak viability of 158.07% at 5 μg/mL. This proliferative rate is comparable to effects reported for standard growth factors like Epidermal Growth Factor (EGF) or therapeutic actives such as allantoin, which typically induce a 120–160% increase in keratinocyte metabolic activity [80]. SFW oleogel significantly outperformed the RBW oleogel (peak 133.04%), suggesting a trade-off between potency and matrix rigidity. The reference system followed the SFW closely, exhibiting a high proliferative potential of 153.70% at 1 μg/mL. This robust response in the SFW oleogel suggests that the carrier oils themselves (camellia/almond), rich in bioactive fatty acids like oleic and linoleic acid, contribute significantly to the regenerative signal, a “vehicle effect” that synergizes with the added essential oils. Meanwhile, HSA oleogel reached its maximal effect at the lowest concentration of 1 μg/mL (130.68%), highlighting the efficiency of its dual-bioactive mechanism.

This stimulatory activity derives from the synergistic phytochemical profile of the entrapped essential oil blend. At sub-cytotoxic levels, the major monoterpenes—specifically geraniol from geranium oil and terpinen-4-ol from tea tree oil—act as signaling molecules. Biochemical studies suggest that these terpenes modulate the mitogen-activated protein kinase (MAPK) pathway, specifically activating ERK1/2, which is a central driver of keratinocyte migration and proliferation [80]. Furthermore, these compounds downregulate nuclear factor-kappa B (NF-κB) and inhibit pro-inflammatory cytokines such as TNF-α, creating a microenvironment that favors division over inflammatory stasis [28]. This finding aligns with the “hormesis in wound healing” concept reviewed by other authors who reported that phytochemicals often induce a 20–60% increase in cell proliferation at low doses via compensatory stress response pathways [103]. Consistent with the prior research, our data suggest a dual-action therapeutic potential for lipid-rich oils: enhancing epidermal regeneration via keratinocyte proliferation and exerting anti-inflammatory effects through the suppression of leukocyte activation [104].

Following the peak proliferation at 5 μg/mL, a distinct downward trend in cell viability was observed initiating at 10 μg/mL across all oleogel formulations. As the concentration increased from 10 to 100 μg/mL, viability values progressively returned toward baseline or dropped slightly below it (e.g., HSA at 100 μg/mL dropped to ~87% at 48 h), marking the end of the proliferative window. This decline signifies the onset of a sub-lethal range where the accumulation of lipophilic active compounds—such as cinnamaldehyde (LogP ≈ 1.9), terpinen-4-ol (LogP ≈ 2.5), and geraniol (LogP ≈ 2.9)—begins to exert mild stress on the cellular machinery. These moderate LogP (partition coefficient) values indicate a high affinity for the lipid bilayer, facilitating rapid membrane partitioning. Mechanistically, this likely involves the generation of low levels of reactive oxygen species (ROS). While low ROS levels act as secondary messengers for proliferation, moderate accumulation can induce oxidative stress that arrests the cell cycle at the G2/M phase [105]. The reference control also exhibited a slight decrease in this range but maintained higher overall viability (e.g., ~117% at 100 μg/mL), further confirming that the sharper decline in the oleogel groups is driven by the increasing load of the entrapped bioactive agents.

The superior proliferative performance of the HSA oleogel at very low doses (1 μg/mL), despite its lower rheological stiffness compared to wax-based systems, suggests a “dual-bioactive” mechanism where the structuring agent itself actively participates in tissue repair. Unlike the inert wax esters in SFW and RBW, 12-hydroxystearic acid is a bioactive lipid. Mechanistic studies indicate that 12-HSA functions as a peroxisome proliferator-activated receptor alpha (PPAR-α) agonist, a pathway critical for normalizing keratinocyte differentiation and stimulating collagen synthesis [106]. Furthermore, recent evidence reveals that 12-HSA induces the secretion of endogenous antimicrobial peptides (AMPs), such as LL-37, by downregulating caspase-8 activity and activating the inflammasome [107]. Additionally, hydroxyl groups at the C-12 position may form transient hydrogen bonds or reversible Schiff base-like interactions with aldehyde groups of cinnamaldehyde, potentially stabilizing volatile payloads more effectively than non-polar waxes [108]. Therefore, the HSA oleogel acts not merely as a passive delivery vehicle but as a synergistic co-active, boosting innate immunity and cellular turnover.

At the highest concentration (500 μg/mL), oleogels crossed a sharp toxicity threshold (Figure 11), causing cell viability to plummet across all essential oil-loaded formulations (20–40%), while the reference control maintained high viability (90–95%). This confirms that the observed cytotoxicity is intrinsic to the bioactive payload. At these high concentrations, cinnamaldehyde functions as a potent electrophile, depleting intracellular glutathione and inducing severe mitochondrial dysfunction via ROS overload [109]. Simultaneously, hydrophobic terpenes partition into the lipid bilayer, increasing permeability and causing leakage of vital ions. However, the extent of this necrotic shift was strictly governed by the oleogel microstructure, exhibiting a strong inverse correlation with the elastic modulus (G′). RBW oleogel, with the highest stiffness (G′ ≈ 57,000 Pa) and crystallinity (ΔH_m_ = 7.79 J/g), exhibited a superior protective capacity (viability ~39% at 24 h). Its dense needle-like network functions as a physical barrier, retarding the diffusion of cytotoxic volatiles—a phenomenon supported by studies on wax-based matrices dampening “burst release” [110]. In contrast, SFW oleogel dropped to ~27% (24 h), suggesting that its “softer” solid-like matrix (G′ ≈ 1.336 Pa) failed to contain the active burst. The resulting biological performance is fundamentally tethered to the rheological profile: the highly rigid, sintered microstructure of the RBW oleogel functions as a restrictive physical barrier that limits the rapid diffusion of cytotoxic volatiles, thereby providing a wider cellular safety margin compared to the softer, more permeable SFW network.

Functionally, these results define a precise therapeutic window (1–5 μg/mL essential oil blend equivalent concentration) for topical application. Within this range, formulations leverage the regenerative properties of essential oils without compromising tissue viability. The data suggest a strategic trade-off in formulation design: while SFW and HSA oleogels offer maximum proliferative potential—ideal for accelerating the closure of non-infected, stalled wounds—RBW oleogel provides a wider safety margin. Its dense network acts as a controlled-release buffer, making RBW the superior candidate for applications requiring higher antimicrobial loading doses where tissue irritation risk must be minimized. Consequently, these oleogels function as bioactive, proliferation-inducing scaffolds, actively supporting the transition from the inflammatory to the proliferative phase of wound healing.

It should be remembered that all optimized oleogels contained 5% (*w*/*w*) essential oil blend concentration, a dosage that targets recalcitrant *Pseudomonas aeruginosa* biofilms. Unlike planktonic bacteria, established biofilms present formidable diffusion barriers requiring aggressive concentrations—up to 5% for constituents like tea tree oil—to penetrate the extracellular polymeric substance matrix [111]. This high loading dose ensures sufficient thermodynamic activity at the biofilm interface while rigorously testing oleogel structuring. Saturating the lipid crystal network establishes a maximal “deposit effect,” transforming the dressing into a long-term reservoir that sustains therapeutic release against resistant pathogens rather than serving as a transient delivery vehicle. Looking forward to clinical translation for non-complicated wound healing scenarios—specifically those lacking severe biofilm involvement—optimizing the balance between antimicrobial efficacy and host tissue safety is crucial. While the 5% concentration used in this study provides aggressive action against resistant strains, a reduced formulation containing a 1–2% essential oil blend would likely emerge as a normal balance for routine applications. The evidence suggests that this concentration range is sufficient to achieve bactericidal activity against common planktonic pathogens, yet it would offer a superior safety margin [112].

A limitation of the present study is the indirect assessment of essential oil release kinetics. The correlation between network density (rheology) and cytotoxicity/antimicrobial efficacy suggests a controlled-release mechanism, where the denser RBW network retards diffusion and the porous SFW network facilitates burst release. However, this structure–function relationship is inferred from biological endpoints. Future studies utilizing Franz diffusion cells or headspace gas chromatography are planned to rigorously quantify the release rates of specific volatile markers (e.g., cinnamaldehyde, terpinen-4-ol) across the different lipid matrices.

## 3. Conclusions

This study successfully establishes that oleogels engineered with sunflower wax (SFW), rice bran wax (RBW), and 12-hydroxystearic acid (HSA) function not merely as inert delivery vehicles, but as active modulators of the cutaneous wound microenvironment. By systematically correlating physicochemical architecture with biological performance, it was demonstrated that the fatty acid profile of the carrier oil is a fundamental determinant of network integrity. Specifically, the high oleic acid content of camellia oil facilitated the formation of superior crystalline networks within RBW systems, whereas the steric hindrance induced by the linoleic acid in almond oil resulted in softer, less cohesive SFW networks. Through multi-objective optimization, three distinct formulations were developed that achieved textural profiles statistically equivalent to the commercial benchmark, validating their mechanical suitability for topical application. Thermal and rheological characterizations revealed a distinct stability hierarchy—RBW > SFW > HSA—that dictates specific functional applications. RBW oleogel, characterized by a high melting point and a rigid, sintered microstructure with high yield stress, emerged as the optimal candidate for heat-stable, protective barrier formulations requiring maximum retention at the skin site. Conversely, the HSA system displayed unique “smart” thixotropic behavior; despite its lower thermal resistance, its self-assembled fibrillar network exhibited rapid structural recovery and strain-hardening ductility. This suggests that HSA formulations are particularly well-suited for cold-processed applications where immediate re-solidification is necessary to prevent dripping and ensure sustained contact. From a biological perspective, the formulations demonstrated robust, broad-spectrum bactericidal activity, achieving a >99% reduction in *Staphylococcus aureus* and *Pseudomonas aeruginosa* viability.

However, a critical hormetic dose–response relationship was identified regarding keratinocyte physiology. While high loading doses effectively eradicated biofilms, a precise therapeutic window was found to significantly stimulate cellular proliferation (1–5 μg/mL essential oil blend equivalent), with SFW and HSA oleogels outperforming the control. A notable trade-off exists in formulation design: the softer SFW and HSA matrices offer superior proliferative signaling, likely due to faster release kinetics or intrinsic gelator bioactivity, whereas the dense crystalline network of RBW provides a protective buffer against cytotoxicity at higher concentrations. Ultimately, these findings underscore the potential of essential oil-enriched oleogels as dual-action therapeutic scaffolds that can shift the wound state from inflammation to proliferation. Future research should now transition from monolayer models to ex vivo stratified skin assays to fully evaluate transdermal flux and validate the immunomodulatory potential of 12-HSA within a complex multicellular environment. While these in vitro physicochemical and antimicrobial findings establish the functional baseline of these lipid scaffolds, future ex vivo and in vivo clinical studies are required to fully validate their prospective application in dynamic skin healing environments.

## 4. Materials and Methods

### 4.1. Essential Oils (EOs)

The bioactive ingredient used in this research consists of an optimized mixture of essential oils, whose development, detailed characterization and experimental validation were reported by our research group in a previous work [113]. This formulation contains 31.7% ginger (*Zingiber officinale*, α-zingiberene: 33.77%), 39.1% cinnamon (*Cinnamomum zeylanicum*, cinnamaldehyde: 77.56%), 14.5% tea tree (*Melaleuca alternifolia*, terpinen-4-ol: 38.38%), and 14.7% geranium (*Pelargonium graveolens*, citronellol: 33.6%) (% *w*/*w*). That prior work employed multi-objective optimization to synergistically maximize bioactivities relevant to cutaneous healing: UV-B photoprotection, cytoprotection via erythrocyte lysis inhibition, and catalase-mediated antioxidant defense. To maintain consistency, the present study used the same essential oils, with previously confirmed identity and purity, acquired from Health & Beauty Natural Oils (HBNO, Chico, CA, USA).

### 4.2. Vegetable Oils

Camellia oil (*Camellia oleifera*) and almond oil (*Prunus Amygdalus Dulcis*) were used as the organic solvents for oleogel formation. Cosmetic oils were acquired from Health & Beauty Natural Oils (HBNO, USA). To verify initial authenticity and quality, fatty acid composition was determined via gas chromatography (GC), following the standard methodology of the Human Food and Nutrition Laboratory at the University of Antioquia [114]. A gas chromatograph (7890, Agilent Technologies, Santa Clara, CA, USA), equipped with a TR-CN100 column and a flame ionization detector (FID), performed the analysis. A 1.0 µL sample was introduced via a split/splitless injector (100:1 ratio) at 260 °C, with a constant helium carrier gas flow of 1.1 mL/min. The oven program began at 90 °C for 7 min, then ramped at 5 °C per minute to 240 °C and held this temperature for 15 min. The detector temperature remained at 300 °C. ChemStation^®^ B.04.03 software (Agilent Technologies, Santa Clara, CA, USA) was used to calculate fatty acid percentages by automatically integrating peak areas and comparing them against pre-established calibration curves. Finally, the Cox index, a crucial predictor of an oil’s oxidative stability based on its fatty acid composition (%), was calculated using Equation (1) [43].

(1) $$Cox\;index=\frac{\left[\left(C16:1\left[\%\right]+C18:1\left[\%\right]+C20:1\left[\%\right]+C24:1\left[\%\right]\right)+10.3\left(C18:2\left[\%\right])+21.6(C18:3\left[\%\right]\right)\right]}{100}$$

### 4.3. Gelling Agents

Three products were evaluated for their potential as oleogelators: (1) 12-hydroxystearic acid (C_18_H_36_O_3_), melting point: 69 °C, acid value: 206 mg KOH/g, saponification index: 208 mg KOH/g, iodine value: ≤0.24 g I_2_/100 g (SpirZon, Clifton, NJ, USA), (2) sunflower wax (*Helianthus annuus*), melting point: 70 °C, acid value: ≤ 8 mg KOH/g, saponification index: 75–95 mg KOH/g, iodine value: ≤12 g I_2_/100 g (Praan Naturals, Oxford, CT, USA), (3) rice bran wax (*Oryza sativa*), melting point: 78 °C, acid value: ≤ 15 mg KOH/g, saponification index: 85.3 mg KOH/g, and iodine value: ≤13 g I_2_/100 g (SpirZon, USA).

### 4.4. Mixture Experimental Design

To evaluate the combined effect of four ingredients—an oil phase (camellia and almond oils), a gelling agent, and a bioactive principle from essential oils—an extreme vertex mixture design was employed. The experimental design, generated by Design Expert 10^®^ software (Stat-Ease, Minneapolis, MN, USA), used a D-optimality criterion to minimize predicted variance across the region. The resulting data were fitted to a quadratic Scheffé polynomial (Equation (2)), a model suitable for mixture experiments where the response (Y) is a function of component proportions (xi) constrained by the relationship 0 ≤ xi ≤ 1 and ∑i = 1qxi = 1 [115]. This model relates the expected response, E(Y), to the component proportions by considering both individual and binary interactions. The linear term coefficients (βi) represent the individual contribution of each component, while the binary interaction term coefficients (βij) quantify synergy (βij > 0) or antagonism (βij < 0). All model coefficients (β) were estimated using least squares regression.

(2) $$E\left(Y\right)=\sum_{i=1}^{q}\beta_{i}x_{i}+\sum_{1\leq i<j\leq q}\beta_{ij}x_{i}x_{j}$$

The experimental design was based on the following component constraints: 1–10% gelling agent, 0–95.3% almond oil, 0–96.7% camellia oil, and 1–5% for the bioactive essential oil principle. Using these constraints, a point exchange algorithm generated 22 experimental runs designed to fit a quadratic Scheffé-type model. This set comprised 18 base model points, 3 replicates to estimate error, and 1 additional point for model validation.

### 4.5. Oleogels Fabrication

Ingredients were weighed (PX323, Ohaus, Parsippany, NJ, USA) to prevent errors from volume and density variations. For each formulation component, determined by experimental design, 10 g per mixture was dosed into 15 mL screw-capped glass vials. Mixtures then underwent hydrothermal heating at 80 ± 1 °C (Precision GP02, Thermo Scientific, Waltham, MA, USA). Continuous mechanical water stirring prevented nonuniform temperature zones that might incompletely solubilize gelling agents. Vials remained capped to prevent evaporative losses. After a 15 min heating time, immediate agitation was performed using a vortex mixer, 300 rpm, 30 s (vortex 3, IKA, Wilmington, NC, USA), and each mixture rested for 24 h at room temperature (24 ± 2 °C) before texture analysis. These heating parameters guaranteed complete gelling agent dissolution and were selected based on the gelator’s physicochemical characteristics and a previous standardizing experimental design. All subsequent analyses were performed in triplicate, and data are presented as the mean ± standard deviation.

### 4.6. Visual Appearance

A qualitative test was performed after the oleogel vials were conditioned for 24 h at 24 ± 2 °C. Each vial was inverted for 30 s, returned to its initial position, and then visually inspected. Based on this inspection, each formulation was classified according to the categories in Table 9 [116].

### 4.7. Textural Analysis

A texture analyzer (TA-XTplus, Stable Micro Systems, Hamilton, MA, USA) with a 5 kg load cell and a P/5S 5 mm spherical-tipped measuring accessory applied downward force to each vial’s oleogel center. Method configuration was as follows: compression force measurement mode; return-to-start option; pre-test speed, 2.0 mm/s; test speed, 1.0 mm/s; post-test speed, 1.0 mm/s; penetration distance, 20 mm; trigger type, Auto–5 g; test mode, Auto; and data acquisition rate, 200 pps. Exponent^®^ 6.1.15 software (Stable Micro Systems, Hamilton, MA, USA) captured data, using a programmed macro to calculate firmness (mN, maximum positive force) and consistency (mN*s, positive force area).

### 4.8. Oleogel Optimization

Oleogel dressings formulation requires balanced textural properties to enhance structural stability, prevent premature disintegration or component release, and ensure a suitable sensory profile during application. An excessively firm or cohesive oleogel may be uncomfortable to apply, whereas a soft or incohesive one can lack stability and efficacy. A commercial product, skin protectant, served as a textural reference; as a dermatological product, it is promoted for its capacity to regenerate and protect the cutaneous barrier, restore skin tissue, reduce inflammation, and strengthen skin’s natural defense mechanisms. Textural analysis values from commercial product provided the benchmark for optimizing mechanical properties (firmness, consistency) of developed oleogels, establishing an objective criterion to evaluate their performance.

Desirability function methodology was employed to simultaneously optimize multiple responses. This approach transforms each individual response (*d_i_*) onto a common desirability scale from zero (least desirable) to one (most desirable), reflecting an optimal range for each variable [117]. A global desirability function, *D(X)*, is calculated as the geometric mean of all transformed responses using Equation (3), where *n* represents the total number of responses. If any single response falls outside its defined desirability range, the global function (*D*) becomes zero, indicating an unacceptable combination of conditions. Each response’s optimization objective can be set to maximize, minimize, or target a specific value. The numerical optimization process seeks factor combinations that maximize this function. A global desirability value of one represents the most favorable outcome, where the resulting responses meet all established criteria. Design-Expert^®^ 10 software (Stat-Ease, Minneapolis, MN, USA) was utilized to identify three (one for each gelling agent) oleogel mixtures with optimal combined performance across the evaluated textural properties. Only the texturally optimized gels underwent subsequent analytical and functional testing.

(3) $$D={\left(d_{1}\cdot d_{2}\cdot \ldots \cdot d_{n}\right)}^{\frac{1}{n}}={\left(\prod_{i=1}^{n}d_{i}\right)}^{\frac{1}{n}}$$

### 4.9. Differential Scanning Calorimetry

Thermal analysis utilized a Q100 DSC equipment (TA Instruments, New Castle, DE, USA). Oleogels were weighed (approximately 10–15 mg) and sealed in an airtight aluminum pan, an empty pan used as a reference for analysis, which occurred under a nitrogen atmosphere at a 600 mL/min flow rate. Samples were cooled from room temperature (22 °C) to 0 °C, subsequently heated from 0 °C to 100 °C at a 10 °C/min rate, held at 100 °C for 10 min, and then cooled from 100 °C to −20 °C at 10 °C/min [118]. An indium standard (156.4 °C < To < 156.8 °C, 28.2 J/g < ΔH< 28.7 J/g) was used to calibrate and verify the equipment. From the resulting thermograms, Universal Analysis^®^ 2000 4.5A software (TA Instruments, New Castle, DE, USA) was used to determine the maximum melting temperature (Tm), melting enthalpy (ΔHm), maximum crystallization temperature (Tc), and crystallization enthalpy (ΔHc).

### 4.10. Thermogravimetric Analysis (TGA)

Oleogels’ thermogravimetric analysis was performed on a TGA Q500 instrument (TA Instruments, New Castle, DE, USA). Samples, held in platinum pans, were heated from 23 °C to 600 °C at a constant rate of 10 °C/min. The analysis occurred under an inert nitrogen atmosphere flowing at 60 mL/min. By evaluating weight percentage change as a temperature function, material’s degradation stages and overall thermal stability were identified.

### 4.11. Fourier Transform Infrared (FTIR) Spectroscopy

FTIR spectra were recorded on a Spectrum Two spectrophotometer (Perkin Elmer, Shelton, CT, USA) equipped with a diamond smart orbit attenuated total reflection (ATR) accessory. A uniform 100-unit force was applied to samples placed directly on the ATR crystal. Each spectrum, collected from 4000 to 450 cm^−1^ at a 4 cm^−1^ resolution, represents 10 accumulated scans performed at 22 °C. All data was processed with Spectrum^®^10 software (Perkin Elmer, Shelton, CT, USA).

### 4.12. Rheological Characterization

Rheological measurements were conducted on a RheoCompass^®^ rheometer (Anton Paar, Graz, Austria), employing a cone-plate geometry (CP50-1, 50 mm diameter, 1° angle). Each sample was equilibrated at the tested temperature (25 °C) for 5 min before tests. For the analysis, a 0.6 mL sample was loaded onto the testing geometry. After first identifying the linear viscoelastic region (LVER) with a preliminary amplitude sweep, a small-amplitude oscillatory shear (SAOS) test was performed over a frequency range of 10^−2^ to 10^2^ rad/s. This test yielded data for the storage modulus (G′), loss modulus (G″), complex viscosity (η*), and the loss tangent (tan δ = G″/G′), which were used to characterize the oleogel’s viscoelastic behavior [119]. Results were fitted to a power law model, G′ = K′ (2π*f*)^n′^, where G′ is the storage modulus (Pa); *f*, frequency (Hz); K′, power law model constant; and n′, frequency index.

### 4.13. Bactericidal Activity

Bactericidal activity was quantified following a standard method [120]. Test suspensions of *Staphylococcus aureus* ATCC 6538 and *Pseudomonas aeruginosa* ATCC 15442 were prepared from fresh Trypticase Soy Agar (TSA) cultures and adjusted to 1 × 10^6^ CFU/mL. In the dilution–neutralization assay, 8.0 mL of the test product was combined with 1.0 mL of bacterial suspension and incubated at 20 °C ± 1 °C; reduction in bacterial viability (Log10 CFU/mL) was quantified after 6 and 12 h of direct contact. The reaction was then stopped by transferring 1.0 mL of the mixture into 9.0 mL of a sterile, validated non-toxic neutralizer (1% *v*/*v* polysorbate 80). Surviving bacteria (CFU) were quantified by plating on TSA, followed by incubation at 36 °C ± 1 °C for 48 h. Viability reduction was determined by comparing the final CFU count with the initial inoculum, with all independent experiments performed in strict triplicate to ensure statistical robustness.

### 4.14. Cell Viability

The viability of immortalized human keratinocytes (HaCaT, CLS 300493; Cell Lines Service GmbH, Germany) was assessed. Cells were maintained in Dulbecco’s Modified Eagle’s Medium (DMEM) supplemented with 10% fetal bovine serum (Welgene, Gyeongsan, Korea), 100 U/mL penicillin, and 100 μg/mL streptomycin, at 37 °C in a 5% CO_2_ atmosphere. HaCaT cells were seeded in 96-well plates at 10,000 cells/well and grown to confluency. The confluent monolayers were then treated with oleogels achieving final essential oil blend equivalent concentrations of 1, 5, 10, 50, 100, and 500 μg/mL for 24 and 48 h. Following treatment, viability was determined by adding 10 μL of WST-8 reagent Cell Counting Kit-8 (Dojindo Molecular Technologies, Rockville, MD, USA) to each well. After a 1 h incubation, optical density (OD) at 450 nm was quantified using a Varioskan Lux spectrophotometer (Thermo Scientific, Waltham, MA, USA). HaCaT cells exposed to dimethyl sulfoxide (DMSO) at concentrations equivalent to those in the test dilutions served as the vehicle control. Blank consisted of cell-free wells containing only culture medium and WST-8 reagent. Results were expressed as the mean ± standard deviation of three independent measurements. Statistical analyses were performed using GraphPad Prism^®^ 8 (GraphPad, Boston, MA, USA). Analysis of variance (ANOVA) and Dunnett’s multiple comparisons test were used to identify significant differences between samples and untreated controls. Cell viability, as a percentage of metabolically viable cells following treatment, was calculated per Equation (4).

(4) $$\%\;\mathrm{viability}=\frac{\mathrm{OD}\;\mathrm{HaCaT}\;\mathrm{exposed}\;\mathrm{to}\;\mathrm{oleogel}\;-\;\mathrm{blank}}{\mathrm{OD}\;\mathrm{HaCaT}\;\mathrm{control}\;-\;\mathrm{blank}\;}\;\times \;100$$

### 4.15. Statistical Analysis

Prior to parametric analysis, the normality of the data distributions was verified. Analysis of variance (ANOVA) followed by Dunnett’s multiple comparisons test was utilized to identify statistically significant differences between the formulated treatments and the untreated controls (Design-Expert 10^®^, Stat-Ease, Minneapolis, MN, USA) with the significance level set at *p* ≤ 0.05. To simplify the final predictive models for each response, non-significant terms were removed, resulting in equations expressed in terms of actual values.

## Figures and Tables

**Table 1:** Fatty acid composition of almond and camellia oils used for oleogel fabrication.

Fatty Acid	Almond		Camellia	
	g/100 g	S.D ±	g/100 g	S.D ±
C16:0 (Palmitic acid)	6.182	0.010	8.476	0.036
C18:0 (Stearic acid)	1.449	0.004	2.093	0.010
Saturated fatty acids (SFAs)	7.632	0.006	10.570	0.047
C16:1 (Palmitoleic acid)	0.488	0.002	0.123	0.005
C18:1n9c (Oleic acid)	66.332	0.025	78.750	0.298
Monounsaturated fatty acids (MUFAs)	66.820	0.027	79.398	0.302
C18:2n6c (Linoleic acid)	23.772	0.017	8.503	0.030
C18:3n3 (α-Linolenic acid)	-	-	0.194	0.004
C20:1n9 (cis-11-Eicosenoic acid)	-	-	0.041	0.057
Polyunsaturated fatty acids (PUFAs)	23.771	0.017	8.739	0.093
Unsaturated fatty acids (UFAs)	90.591	0.045	88.138	0.396
TOTAL FAT	98.223	0.016	98.708	0.443
UFA/SFA	11.870	0.003	8.338	0.001
O/L	2.790	0.001	9.261	0.002
MUFA/SFA	8.756	0.003	7.512	0.004
PUFA/SFA	3.115	0.002	0.827	0.005

**Table 2:** D-optimal mixture design and experimental results for the oleogels’ textural parameters.

Mixture	Ingredients (Fraction)				SFW			RBW			HSA		
	G.A	A.O	C.O	E.O.B	Va	Firm	Cons	Va	Firm	Cons	Va	Firm	Cons
	*x*_1_	*x*_2_	*x*_3_	*x*_4_		mN	mN*s		mN	mN*s		mN	mN*s
1	0.100	0.000	0.850	0.050	Fr	931.05 ± 44.22	4739.36 ± 103.79	Fr	405.91 ± 18.27	2219.18 ± 95.42	Fr	154.68 ± 6.03	750.93 ± 25.53
2	0.010	0.653	0.327	0.010	Sf	2.97 ± 0.08	41.86 ± 1.13	Fr	1.29 ± 0.06	16.93 ± 0.44	Sl	0	0
3	0.070	0.880	0.000	0.050	Fr	296.24 ± 11.26	1474.87 ± 57.52	Fr	285.02 ± 13.11	1111.50 ± 47.24	Fr	6.47 ± 0.28	104.16 ± 2.60
4	0.010	0.653	0.327	0.010	Sf	1.98 ± 0.05	27.08 ± 1.30	Sf	1.37 ± 0.03	17.84 ± 0.84	Sl	0	0
5	0.010	0.953	0.000	0.037	Sf	1.83 ± 0.04	13.99 ± 0.31	Sf	0.91 ± 0.04	10.82 ± 0.41	Sl	0	0
6	0.077	0.229	0.654	0.040	Fr	568.89 ± 26.17	2561.41 ± 105.02	Fr	318.06 ± 8.59	2213.74 ± 55.34	Fr	11.59 ± 0.49	197.42 ± 9.48
7	0.070	0.880	0.000	0.050	Fr	413.02 ± 14.87	1716.49 ± 77.24	Fr	284.94 ± 13.11	1568.12 ± 61.16	Fr	28.61 ± 0.69	126.18 ± 4.54
8	0.100	0.000	0.890	0.010	Fr	971.75 ± 36.93	4065.77 ± 187.03	Fr	528.85 ± 25.38	1879.08 ± 41.34	Fr	137.44 ± 6.73	741.09 ± 37.05
9	0.055	0.458	0.457	0.030	Fr	371.93 ± 14.13	1660.02 ± 33.20	Fr	127.69 ± 4.47	610.70 ± 29.32	Fr	5.82 ± 0.28	88.67 ± 3.81
10	0.100	0.870	0.000	0.030	Fr	781.22 ± 22.66	4647.67 ± 199.85	Fr	605.92 ± 26.66	3775.07 ± 181.20	Fr	131.30 ± 5.65	743.15 ± 34.19
11	0.055	0.458	0.457	0.030	Fr	208.34 ± 5.00	930.83 ± 45.61	Fr	224.91 ± 5.62	707.41 ± 14.86	Fr	5.31 ± 0.26	88.14 ± 2.81
12	0.100	0.283	0.567	0.050	Fr	926.54 ± 20.38	4281.01 ± 141.27	Fr	508.17 ± 15.25	2608.69 ± 122.61	Fr	132.68 ± 3.58	700.80 ± 23.83
13	0.077	0.674	0.229	0.020	Fr	581.19 ± 16.27	2433.11 ± 116.80	Fr	539.56 ± 22.66	2380.02 ± 59.50	Fr	9.73 ± 0.38	161.35 ± 7.91
14	0.010	0.000	0.967	0.023	Sf	1.14 ± 0.05	24.24 ± 1.16	Sf	1.75 ± 0.08	13.65 ± 0.44	Sl	0	0
15	0.032	0.229	0.719	0.020	Fr	106.76 ± 3.74	448.78 ± 15.26	Fr	9.31 ± 0.21	76.15 ± 3.73	Sf	1.69 ± 0.04	22.90 ± 0.53
16	0.100	0.870	0.000	0.030	Fr	727.13 ± 29.81	4346.94 ± 95.63	Fr	610.47 ± 25.64	3794.18 ± 117.62	Fr	138.82	1019.98 ± 45.90
17	0.010	0.953	0.000	0.037	Sf	1.83 ± 0.06	14.59 ± 0.50	Sf	1.14 ± 0.05	13.68 ± 0.47	Sl	0	0
18	0.100	0.567	0.283	0.050	Fr	669.93 ± 22.11	3500.02 ± 133.00	Fr	607.67 ± 25.52	3346.63 ± 154.00	Fr	113.67 ± 2.50	719.91 ± 17.28
19	0.010	0.313	0.627	0.050	Fr	89.32 ± 1.97	28.91 ± 1.10	Sf	1.83 ± 0.05	16.13 ± 0.32	Sl	0	0
20	0.010	0.313	0.627	0.050	Fr	87.51 ± 4.42	31.16 ± 0.81	Sf	1.64 ± 0.03	15.54 ± 0.43	Sl	0	0
21	0.055	0.458	0.457	0.030	Fr	89.36 ± 4.38	1096.01 ± 24.11	Fr	124.07 ± 3.97	533.87 ± 25.63	Fr	5.10 ± 0.24	74.79 ± 3.59
22	0.055	0.935	0.000	0.010	Fr	90.59 ± 2.63	811.40 ± 29.21	Fr	188.63 ± 9.05	759.46 ± 28.10	Sf	4.56 ± 0.10	74.47 ± 2.61

Gelling agent: G.A; Almond oil: A.O; Camellia oil: C.O; Essential oil blend: E.O.B; SFW: Sunflower wax; RBW: Rice bran wax; HSA: 12-hydroxystearic acid; Va: Visual appearance; In: Insoluble; Sl: Solubilized; Sf: Soft; Fr: Firm; Firmness: Firm; Consistency: Cons. Mixtures with zero (0) value were liquids.

**Table 3:** Analysis of variance (ANOVA) for the textural properties of oleogels with different gelling agents.

		Firmness					Consistency				
	Source	Sum Squares	df	Mean Square	F- Value	*p*- Value	Sum Squares	df	Mean Square	F- Value	*p*- Value
**Sunflower wax**	Model	2.453 × 10^6^	9	2.726 × 10^5^	33.84	<0.0001	6.369 × 10^7^	9	7.077 × 10^6^	94.43	<0.0001
	Mixture	2.273 × 10^6^	3	7.578 × 10^5^	94.10	<0.0001	5.842 × 10^7^	3	1.947 × 10^7^	259.85	<0.0001
	x_1_*x_2_	1.346 × 10^5^	1	1.346 × 10^5^	16.71	0.0015	4.141 × 10^6^	1	4.141 × 10^6^	55.25	<0.0001
	x_1_*x_3_	1.276 × 10^5^	1	1.276 × 10^5^	15.85	0.0018	4.075 × 10^6^	1	4.075 × 10^6^	54.37	<0.0001
	x_1_*x_4_	10,160.34	1	10,160.34	1.26	0.2833	53,237.96	1	53,237.96	0.7104	0.4158
	x_2_*x_3_	1391.89	1	1391.89	0.1728	0.6850	18,208.10	1	18,208.10	0.2430	0.6310
	x_2_*x_4_	2.10	1	2.10	0.0003	0.9874	4.345 × 10^5^	1	4.345 × 10^5^	5.80	0.0330
	x_3_*x_4_	2.04	1	2.04	0.0003	0.9876	4.598 × 10^5^	1	4.598 × 10^5^	6.13	0.0291
	Lack fit	48,108.30	5	9621.66	1.39	0.3340	5.325 × 10^5^	5	1.065 × 10^5^	2.03	0.1906
	**Total**	2.550 × 10^6^	21				6.459 × 10^7^	21			
**Rice bran wax**	Model	1.735 × 10^6^	9	1.928 × 10^5^	163.77	<0.001	8.049 × 10^7^	9	8.943 × 10^6^	32.70	<0.001
	Mixture	1.473 × 10^6^	3	4.910 × 10^5^	417.03	<0.001	5.671 × 10^7^	3	1.890 ± 10^7^	69.12	<0.001
	x_1_*x_2_	1.577 10^5^	1	1.577 × 10^5^	133.95	<0.001	1.183 × 10^7^	1	1.183 × 10^7^	43.26	<0.001
	x_1_*x_3_	1.711 × 10^5^	1	1.711 × 10^5^	145.31	<0.001	1.304 × 10^7^	1	1.304 × 10^7^	47.67	<0.001
	x_1_*x_4_	685.50	1	685.50	0.58	0.462	1.047 × 10^6^	1	1.047 × 10^6^	3.83	0.071
	x_2_*x_3_	6419.35	1	6419.35	5.45	0.371	41,251.31	1	41,251.31	0.15	0.706
	x_2_*x_4_	23,745.49	1	23,745.49	20.17	0.784	3.845 × 10^6^	1	3.845 × 10^6^	14.06	0.851
	x_3_*x_4_	23,961.44	1	23,961.44	20.35	0.729	3.953 × 10^6^	1	3.953 × 10^6^	14.45	0.595
	Lack fit	7870.98	5	1574.20	1.76	0.234	3.010 × 10^6^	5	6.021 × 10^5^	15.50	0.101
	**Total**	1.750 × 10^6^	21				8.377 × 10^7^	21			
**Hydroxystearic acid**	Model	71,536.81	9	7948.53	25.62	<0.001	2.302 × 10^6^	9	2.558 × 10^5^	30.90	<0.001
	Mixture	50,148.16	3	16,716.05	53.88	<0.001	1.716 × 10^6^	3	5.720 × 10^5^	69.10	<0.001
	x_1_*x_2_	16,472.22	1	16,472.22	53.10	<0.001	5.114 × 10^5^	1	5.114 × 10^5^	61.77	<0.001
	x_1_*x_3_	16,266.14	1	16,266.14	52.43	<0.001	5.149 × 10^5^	1	5.149 × 10^5^	62.20	<0.001
	x_1_*x_4_	356.48	1	356.48	1.15	0.348	1551.16	1	1551.16	0.18	0.678
	x_2_*x_3_	276.77	1	276.77	0.89	0.335	176.66	1	176.66	0.02	0.883
	x_2_*x_4_	16.49	1	16.49	0.05	0.816	12,194.25	1	12,194.25	1.47	0.242
	x_3_*x_4_	19.97	1	19.97	0.06	0.840	12,658.18	1	12,658.18	1.53	0.239
	Lack fit	3448.69	5	689.74	17.61	0.108	60,686.58	5	12,137.32	2.20	0.167
	**Total**	75,259.59	21				2.402 × 10^6^	21			

Gelling agent: x_1_; Almond oil: x_2_; Camellia oil: x_3_; Essential oil blend: x_4._

**Table 4:** Predictive models for the textural properties of oleogels with different gelling agents.

	Parameter	x_1_	x_2_	x_3_	x_4_	x_1_*x_2_	x_1_*x_3_	R^2^
SWF	Firmness	95,685.73	−78.84	−64.51	4572.74	−96,746.20	−94,509.67	0.962
	Consistency	522,991.87	−115.23	−800.89	−808,661.40	−536,625.89	−534,017.87	0.986
RBW	Firmness	63,383.120	−71.042	−41.832	−3045.659	−61,844.137	−63,646.771	0.967
	Consistency	447,411.45	−423.62	−677.76	−749,521.46	−456,217.46	−474,442.21	0.971
HSA	Firmness	31,441.94	34.3	25.01	−5305.27	−33,846.01	−33,741.68	0.913
	Consistency	177,168.56	62.34	27.65	−135,449.73	−188,576.63	−189,835.77	0.927

SFW: Sunflower wax; RBW: Rice bran wax; HSA: 12-hydroxystearic acid; Gelling agent: x_1_; Almond oil: x_2_; Camellia oil: x_3_; Essential oil blend: x_4._

**Table 5:** Optimized oleogels formulations and comparisons of the textural properties studied.

Oleogel	Optimized Formulations					Validation (n = 3)		Control (n = 3)		*p*-Value	
	x_1_	x_2_	x_3_	x_4_	*D*	Firm (mN)	Cons (mN*s)	Firm (mN)	Cons (mN*s)	Firm	Cons
**SFW**	0.012	0.554	0.384	0.050	0.969	5.242 ± 0.229	82.020 ± 0.450	5.117 ± 0.351	80.385 ± 1.404	0.125	0.571
**RBW**	0.010	0.280	0.660	0.050	0.986	5.670 ± 0.285	81.466 ± 2.381			0.553	0.803
**HSA**	0.067	0.680	0.204	0.050	0.958	5.185 ± 0.177	79.815 ± 0.567			0.068	0.634

SFW: Sunflower wax; RBW: Rice bran wax; HSA: 12-hydroxystearic acid; Gelling agent: x_1_; Almond oil: x_2_; Camellia oil: x_3_; Essential oil blend: x_4_; D: Desirability. Firmness: Firm; Consistency: Cons; *p*-value: Dunnett test, n: Replicates.

**Table 6:** Thermal parameters of optimized oleogels formulations.

Cycle	Oleogels	Ts (°C)	Tp (°C)	Te (°C)	Thermal Event Range (°C)	ΔH (J/g)
Heating	SFW	35.36 ± 0.41	50.62 ± 0.3	55.26 ± 0.22	19.90 ± 0.47	3.28 ± 0.15
	RBW	46.99 ± 0.73	60.09 ± 0.5	62.24 ± 0.68	15.25 ± 1.00	7.79 ± 0.74
	HSA	6.08 ± 0.55	22.47 ± 0.6	25.93 ± 0.81	19.85 ± 0.98	12.25 ± 0.77
Cooling	SFW	67.46 ± 0.37	57.79 ± 0.4	38.21 ± 0.63	29.25 ± 0.73	3.44 ± 0.63
	RBW	76.53 ± 0.52	68.41 ± 0.2	53.93 ± 0.75	22.60 ± 0.91	6.58 ± 0.48
	HSA	48.94 ± 0.49	40.87 ± 0.8	27.53 ± 0.86	21.41 ± 0.99	6.79 ± 0.84

SFW: Sunflower wax; RBW: Rice bran wax; HSA: 12-hydroxystearic acid; Temperatures: onset (Ts), peak (Tp), end (Te).

**Table 7:** Rheological parameters of optimized oleogels formulations (amplitude sweeps).

Oleogel	Linear Viscoelastic Range				Yield Point		Flow Point	
	Strain (%)	G′ (Pa)	G″ (Pa)	tan(δ)	Strain (%)	Stress (Pa)	Strain (%)	Stress (Pa)
SFW	0.000215 ± 0.000010	24,350.30 ± 1106	7537.17 ± 325	0.3120 ± 0.0150	0.000450 ± 0.000027	14.8534 ± 0.7567	0.00580 ± 0.00030	22.607 ± 1.256
RBW	0.000205 ± 0.000009	56,916.00 ± 3252	16,288.20 ± 1007	0.2862 ± 0.0209	0.000335 ± 0.000014	20.9080 ± 0.9033	0.00650 ± 0.00043	28.430 ± 0.934
HSA	0.000197 ± 0.000006	21,144.00 ± 1179	5488.30 ± 281	0.2596 ± 0.0107	0.000505 ± 0.000031	10.7680 ± 0.4762	0.00748 ± 0.00034	89.510 ± 5.758

**Table 8:** Power law parameters of optimized oleogels formulations.

Oleogel	Storage Modulus			Loss Modulus			Loss Factor			Viscosity		
	(G′) (Pa)			(G″) (Pa)			(tan δ)			(Pa·s)		
	K	n	R^2^	K	n	R^2^	K	n	R^2^	K	n	R^2^
SFW	14,520.05 ± 850.62	0.141 ± 0.01	0.991	4501.72 ± 210.49	0.122 ± 0.01	0.985	0.310 ± 0.01	0.017 ± 0.001	0.928	185,934 ± 9560	−1.250 ± 0.05	0.994
RBW	31,330.48 ± 1275.87	0.092 ± 0.03	0.995	8960.80 ± 408.82	0.115 ± 0.04	0.984	0.286 ± 0.02	0.013 ± 0.003	0.919	493,015 ±19,720	−1.594 ± 0.08	0.992
HSA	8225.28 ± 338.31	0.044 ± 0.01	0.993	2130.30 ± 115.31	0.055 ± 0.02	0.991	0.259 ± 0.01	0.019 ± 0.002	0.945	11,186 ± 671	−0.881 ± 0.05	0.987

**Table 9:** Qualitative classification framework for oleogel visual appearance.

Classification	Description	Flow Behavior	Technical Interpretation
Insoluble	Gelling agent fails to dissolve	Visible separated solid phase	Incompatibility with the oleaginous medium
Solubilized	Agent dissolves but forms no gel structure	Free-flowing, non-cohesive liquid	Absence of a three- dimensional network
Soft	Semi-solid structure with slow uniform fluidity	Slow, cohesive, and controlled flow	Forms a gel suitable for topical application
Firm	Rigid structure; resists flow without external force	No flow when vial is inverted	Excessive rigidity hinders spreadability and removal

## Data Availability

The data supporting the findings of this study are available from the corresponding author upon reasonable request.
